# PRMT5 promotes ovarian cancer growth through enhancing Warburg effect by methylating ENO1

**DOI:** 10.1002/mco2.245

**Published:** 2023-03-28

**Authors:** Fei Xie, Han Zhang, Kongkai Zhu, Cheng‐Shi Jiang, Xiaoya Zhang, Hongkai Chang, Yaya Qiao, Mingming Sun, Jiyan Wang, Mukuo Wang, Junzhen Tan, Tao Wang, Lianmei Zhao, Yuan Zhang, Jianping Lin, Chunze Zhang, Shuangping Liu, Jianguo Zhao, Cheng Luo, Shuai Zhang, Changliang Shan

**Affiliations:** ^1^ State Key Laboratory of Medicinal Chemical Biology, College of Pharmacy and Tianjin Key Laboratory of Molecular Drug Research Nankai University Tianjin China; ^2^ Advanced Medical Research Institute Shandong University Jinan China; ^3^ School of Biological Science and Technology University of Jinan Jinan China; ^4^ Biomedical Translational Research Institute Jinan University Guangzhou Guangdong China; ^5^ School of Integrative Medicine Tianjin University of Traditional Chinese Medicine Tianjin China; ^6^ Tianjin Key Laboratory of human development and reproductive regulation Tianjin Central Hospital of Obstetrics and Gynecology Tianjin China; ^7^ Research Center The Fourth Hospital of Hebei Medical University Shijiazhuang Hebei China; ^8^ The Sixth Affiliated Hospital of Guangzhou Medical University Qingyuan Guangdong China; ^9^ Department of Colorectal Surgery, Tianjin Union Medical Center Nankai University Tianjin China; ^10^ Department of Pathology, Medical School Dalian University Dalian Liaoning China; ^11^ State Key Laboratory of Drug Research Shanghai Institute of Materia Medica Chinese Academy of Sciences Shanghai China

**Keywords:** alpha‐enolase (ENO1), glycolysis flux, ovarian cancer, protein arginine methyltransferase 5 (PRMT5), symmetric dimethylation of arginine (SDMA)

## Abstract

Protein arginine methyltransferase 5 (PRMT5) is a major type II enzyme responsible for symmetric dimethylation of arginine (SDMA), and plays predominantly roles in human cancers, including in ovarian cancer. However, the exactly roles and underlying mechanisms of PRMT5 contributing to the progression of ovarian cancer mediated by reprogramming cell metabolism remain largely elusive. Here, we report that PRMT5 is highly expressed and correlates with poor survival in ovarian cancer. Knockdown or pharmaceutical inhibition of PRMT5 is sufficient to decrease glycolysis flux, attenuate tumor growth, and enhance the antitumor effect of Taxol. Mechanistically, we find that PRMT5 symmetrically dimethylates alpha‐enolase (ENO1) at arginine 9 to promotes active ENO1 dimer formation, which increases glycolysis flux and accelerates tumor growth. Moreover, PRMT5 signals high glucose to increase the methylation modification of ENO1. Together, our data reveal a novel role of PRMT5 in promoting ovarian cancer growth by controlling glycolysis flux mediated by methylating ENO1, and highlights that PRMT5 may represent a promising therapeutic target for treating ovarian cancer.

## Graphical abstract

PRMT5 promotes ovarian cancer growth and Taxol response through enhancing glycolysis pathway, which mediated by regulating the symmetric dimethylation of arginine (SDMA) modification of ENO1.

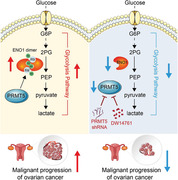

## 1 INTRODUCTION

Ovarian cancer is one of the common malignant tumors of female reproductive system, and its incidence and mortality rates are among the top three in female cancers, which seriously threaten women's life and health.^1^, ^2^ Due to early‐stage asymptomatic and limited screening methods for ovarian cancer, the early diagnosis of this cancer is relatively difficult. Approximately 60% of ovarian cancer patients are already in the advanced stage at the time of diagnosis. Combination of Taxol and platinum is the first‐line chemotherapy for ovarian cancer.^3^, ^4^ However, about 80% of patients have successive relapses after treatment, resulting in a gradual shortening of the treatment‐free period, which is likely to make patients resistant to drugs. Therefore, diagnosed late and drug resistance are two important factors leading to the high morbidity and mortality of ovarian cancer. Thus, it is urgent to explore molecular mechanisms underlying ovarian cancer carcinogenesis, and may help to develop new targeted therapies for treating ovarian cancer.

Protein arginine methylation modification, catalyzed by protein arginine methyltransferases (PRMTs), has been identified as one of the most common posttranslational modifications (PTMs), which regulates extensive cellular processes and is recognized as driver for human diseases including cancer pathogenesis.^5^, ^6^, ^7^ PRMT5 is a type‐II PRMT that catalyzes symmetric dimethylation of arginine (SDMA) of protein substrates.^8^ It is composed of 637 amino acids (aa) and participates in various biological processes in the human body, including the production of ribosomes, Golgi assembly, cell differentiation, cell proliferation, apoptosis, and embryonic cell development.^7^, ^9^, ^10^ In addition to regulating the gene transcription by catalyzing the dimethylation of histone arginine residues (H2AR3, H3R8, and H4R3),^11^ PRMT5 also methylates the nonhistone arginine residues, such as tumor suppressor p53 and sterol regulatory element‐binding transcription factors sterol regulatory element‐binding protein (SREBP), to regulate the biological process of tumor cells.^12^, ^13^ PRMT5 is not only an important epigenetic enzyme, but also an important oncogene that mediates the occurrence and development of solid tumors.^14^ It is highly expressed in many solid tumors (such as ovarian cancer, breast cancer, colorectal cancer, gastric cancer, melanoma, and malignant gliomas) and leukemia or lymphoid cancer, which is directly related to the malignant degree of tumor, tumor progression, clinical prognosis, and survival rate of patients with certain tumor recurrences.^14^, ^15^, ^16^, ^17^ Nevertheless, more research is needed to explore the role of PRMT5 in cell metabolism, tumor growth, and Taxol responses of ovarian cancer.

In the 1920s, Otto Warburg discovered that tumor cells ferment glucose and preferentially select glycolysis pathway for adenosine triphosphate (ATP) production, even under aerobic conditions.^18^, ^19^ Alpha‐enolase (ENO1), the key metabolic enzyme in glycolysis pathway, catalyzes 2‐phosphoglycerate (2‐PG) to phosphoenolpyruvate (PEP) in its active state of the dimer.^20^, ^21^ Currently, extensive studies have shown that ENO1 is regulated by different mechanisms, thereby regulating the progress of cancer by affecting the glycolysis pathway. In addition to the transcriptional regulation,^22^, ^23^ mRNA alternative splicing,^24^ and protein–protein interactions,^25^, ^26^ the PTMs also play an important role in the regulation of ENO1. For example, ULK1/2 reduces its enzymatic activity by phosphorylating ENO1 at Ser115 and Ser282.^27^ In colorectal cancer, the absence of EP300 inhibits the enzyme activity of ENO1 by reducing the level of lysine 2‐hydroxyisobutyrylation (Khib) on ENO1, thereby affecting glycolysis.^28^ There is also evidence that other modifications including O‐GlcNAcylation, acetylation, and methylation regulate the function of ENO1, and the level of these modifications in cancer cells is significantly higher than that in normal cells.^29^, ^30^ However, the role of symmetric dimethyl arginine (SDMA) modifications on ENO1 in regulating cell metabolism, tumor growth, and chemotherapy responses of ovarian cancer remains largely unknown.

The goal of this study was to explore the role and mechanism of PRMT5 in cell metabolism, cancer progression, and asses its potential as a therapeutic target. In this study, we found that PRMT5 is upregulated in ovarian cancer and promotes ovarian cancer cell glycolysis flux, tumor growth, and Taxol response. Further, we revealed that PRMT5 promotes glycolysis flux by symmetrically dimethylating ENO1, leading to the active ENO1 dimer formation. Given the important in ovarian cancer progression mediated by glycolysis flux, we applied previously designed and synthesized PRMT5 inhibitors, which block glycolysis flux, tumor growth, and enhance antitumor effects of Taxol in ovarian cancer. Taken together, our results showed that the PRMT5‐ENO1 axis drives ovarian cancer progression, and further suggested that this axis may be a novel therapeutic target for treating ovarian cancer.

## 2 RESULTS

### 2.1 PRMT5 is increased in ovarian cancer and important for ovarian cancer growth

**FIGURE 1:**
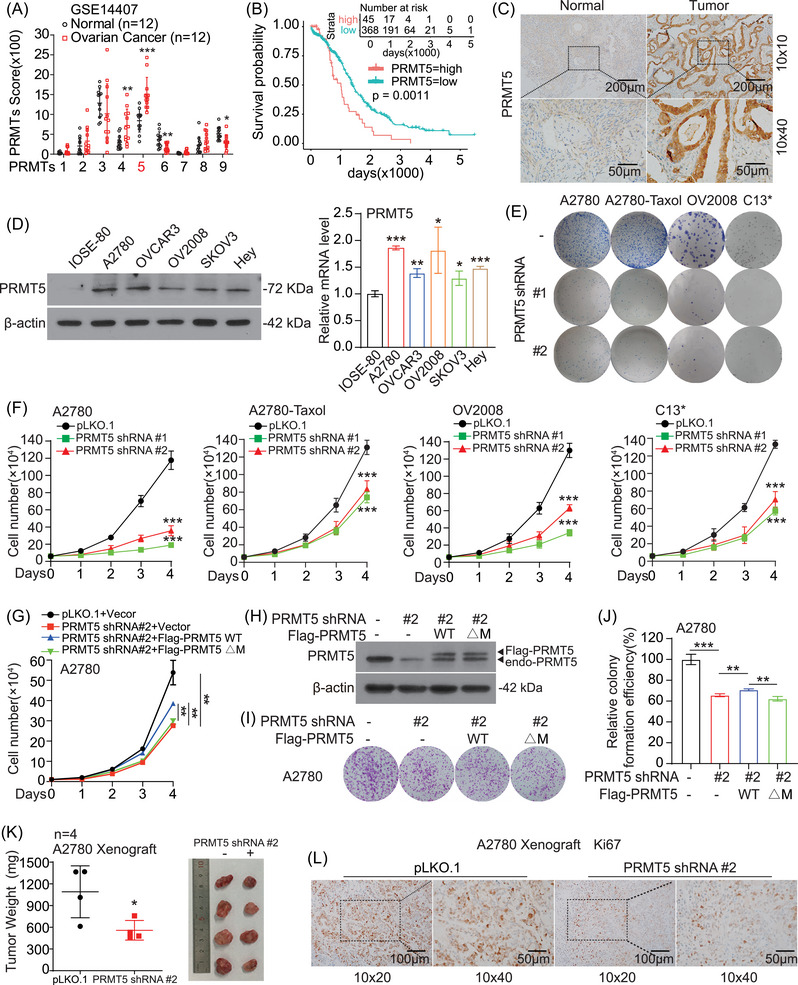
PRMT5 is increased in ovarian cancer and important for ovarian cancer growth. (A) The expressions of PRMTs were examined based on GEO databases (GSE14407). (B) Kaplan–Meier analysis of overall survival was performed on the basis of PRMT5 expression level in ovarian cancer patients using TCGA database. (C) IHC was performed to detect the PRMT5 protein level in clinical sample tissues of ovarian cancer (Cohort 2: ovarian cancer tissues [T, n = 39], normal tissues [N, n = 22]). (D) The protein and mRNA levels of PRMT5 were detected in various human ovarian cancer cells and normal cell. (E) Colony formation assay was examined in various ovarian cancer cells with PRMT5 stable knockdown. (F) The cell proliferation was determined by cell number counting in ovarian cancer cells with stable knockdown of PRMT5. (G) Cell proliferation was determined by cell number counting in PRMT5 knockdown A2780 cells, which exogenous with Flag‐PRMT5 WT or inactivating mutant (ΔM). (H) The expression levels of PRMT5 were detected in PRMT5 knockdown A2780 cells, which exogenously express Flag‐PRMT5 WT or inactivating mutant (ΔM). (I) Colony formation assay was examined in PRMT5 knockdown A2780 cells, which is exogenous with Flag‐PRMT5 WT or inactivating mutant (ΔM). (J) Statistical analyses of colony formation in Figure 1I were performed. (K) Tumor mass was examined in xenograft nude mice bearing A2780 cells tumor with PRMT5 knockdown and control vector (n = 4). And all tumors in xenograft nude mouse are shown. (L) IHC was performed to detect the Ki67 level in representative tumors. (All error bars, mean values  ±  SD, p values were determined by unpaired two‐tailed Student's t test of n  =  3 independent biological experiments. *p < 0.05; **p < 0.01; ***p < 0.001).

To identify the key drive epigenetic regulators in ovarian cancer progression, we analyzed the expression levels of PRMT family (PRMT1‐PRMT9) based on GEO database (GSE14407). We found that the levels of PRMT5 were evidently increased in ovarian cancer (Figure 1A). Furthermore, we found that PRMT5 was also highly expressed in ovarian cancer tissues based on four other GEO databases (GSE66957, GSE120259, GSE12470, and GSE38666) (Figure S1A). In addition, the expression of PRMT5 was also elevated in Grade III relative to Grade I/II (GSE14764) (Figure S1B). Lastly, the elevated expression of PRMT5 predicted the lower survival probability in ovarian cancer (Figure 1B and Figure S1C). Thus, the following investigations were focused on the mechanism and role of PRMT5 in promoting ovarian cancer.

To explore the clinical relevance of our findings, we performed immunohistochemistry (IHC) staining to determine the PRMT5 protein levels in clinical sample tissues of ovarian cancer (Cohort 1: ovarian cancer tissues [T, n = 39], normal tissues [N, n = 22]). We found that the expression of PRMT5 was positive in 22 (56.4%) of the 39 ovarian cancer patients (Table S1), and it was significantly higher in ovarian cancer than normal tissues (Figure 1C and Figure S1D). In another clinical sample library of ovarian cancer patients, we observed that there is no correlation between tumor grade and PRMT5 levels (Table S2, ovarian cancer tissues, Cohort 2: n = 65). However, after analyzing 32 patients who provided prognostic information in this library, we found that patients with high PRMT5 expression predicted poor prognosis in ovarian cancer (Figure S1E). Next, we checked the expression of PRMT5 in various human ovarian cancer cells (A2780, OV2008, OVCAR3, SKOV3, and Hey‐T30 cells). We found that the protein level of PRMT5 in ovarian cancer cells was significantly higher than that in normal proliferative cells (IOSE‐80 cells), although the mRNA level was only slightly increased (Figure 1D). What is more, the PRMT5 expression was higher in Taxol‐resistant ovarian cancer cell lines (A2780‐Taxol, C13*, and OVCAR3‐Taxol) than that in their corresponding parental cell lines (A2780, OV2008, and OVCAR3) (Figure S1F). Together, our results demonstrated that the expression of PRMT5 was highly increased in ovarian cancer and was associated with the overall survival of patients, suggesting that PRMT5 is a promising therapeutic target in ovarian cancer.

To explore the functional role of PRMT5 in ovarian cancer, we generated PRMT5 stable knockdown cell line by specific short hairpin RNAs (shRNAs), and found that knockdown PRMT5 dramatically decreased A2780, A2780‐Taxol, OV2008, and C13* cells proliferation by colony formation assay and cell number counting assay (Figure 1E and F and Figure S1G). The decreased cell proliferation was rescued by exogenous expression of PRMT5 wild type (WT), but not the inactivating mutant of PRMT5 (ΔM), which lacked the sequence GAGRG at 365−369 amino acid (aa)^8^, ^13^ (Figure 1G–J). These findings showed that PRMT5 promotes ovarian cancer cell proliferation in vitro. Next, we explore the role of PRMT5 in A2780 cells‐derived xenograft (CDX) mouse model and found that knockdown PRMT5 showed a slower growth rate compared with control group (Figure 1K and L and Figure S1H). Together these data demonstrated that PRMT5 plays an important role in promoting ovarian cancer growth.

### 2.2 PRMT5 promotes ovarian cancer cell glycolysis flux

**FIGURE 2:**
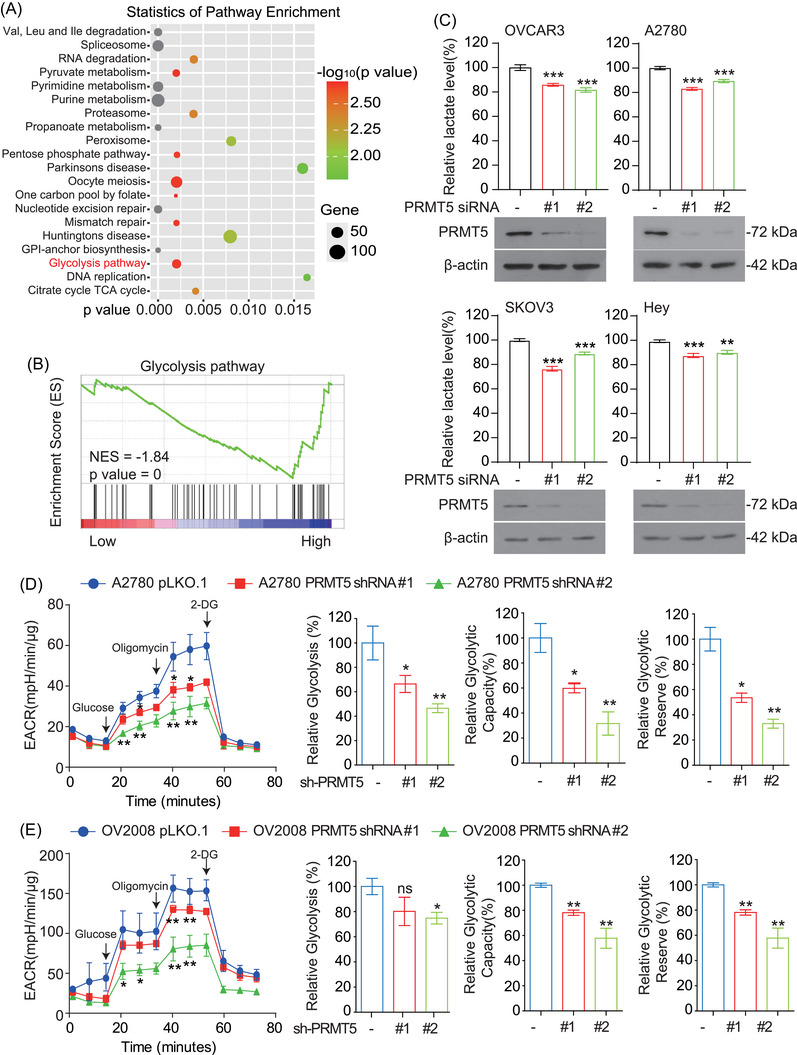
PRMT5 promotes ovarian cancer cell glycolysis flux. (A) KEGG pathway analysis was performed in ovarian cancer database grouped by PRMT5 expression. (B) Glycolysis pathway was enriched in KEGG analysis. (C) The lactate production was detected in various ovarian cancer cells with PRMT5 knockdown by siRNA. (D) The extracellular acidification rate (ECAR) was measured in shRNA‐mediated PRMT5 knockdown A2780 cells. (2‐DG, 2‐Deoxy‐D‐glucose, the inhibitor of glycolytic pathway). (E) The ECAR was measured in OV2008 cells with shRNA‐mediated PRMT5 knockdown. (All error bars, mean values  ±  SD, p values were determined by unpaired two‐tailed Student's t test of n  =  3 independent biological experiments. *p < 0.05; **p < 0.01; ***p < 0.001).

We next explored the possible underlying signaling mechanisms of PRMT5‐driven ovarian cancer cell growth. First, the data of ovarian cancer patients downloaded from the cBioPortal database were grouped according to the expression level of PRMT5.^31^, ^32^ Thereafter, Kyoto Encyclopedia of Genes and Genomes (KEGG) pathway analysis showed that the top 20 enriched pathways included the glycolysis pathway (Figure 2A and B). To further identify the role of glycolysis pathway as the downstream effector of PRMT5 in promoting ovarian cancer development, we explored the effects of PRMT5 on glycolysis pathway. We found that knockdown of PRMT5 with siRNA decreased lactate production in ovarian cancer cells (Figure 2C). In addition, targeting PRMT5 with shRNA reduced extracellular acidification rate (ECAR) (Figure 2D and E) in ovarian cancer cells. These data together suggested that PRMT5 plays a vital role in ovarian cancer cells by regulating glycolysis flux.

### 2.3 PRMT5 increases ENO1 activity by arginine methylation

**FIGURE 3:**
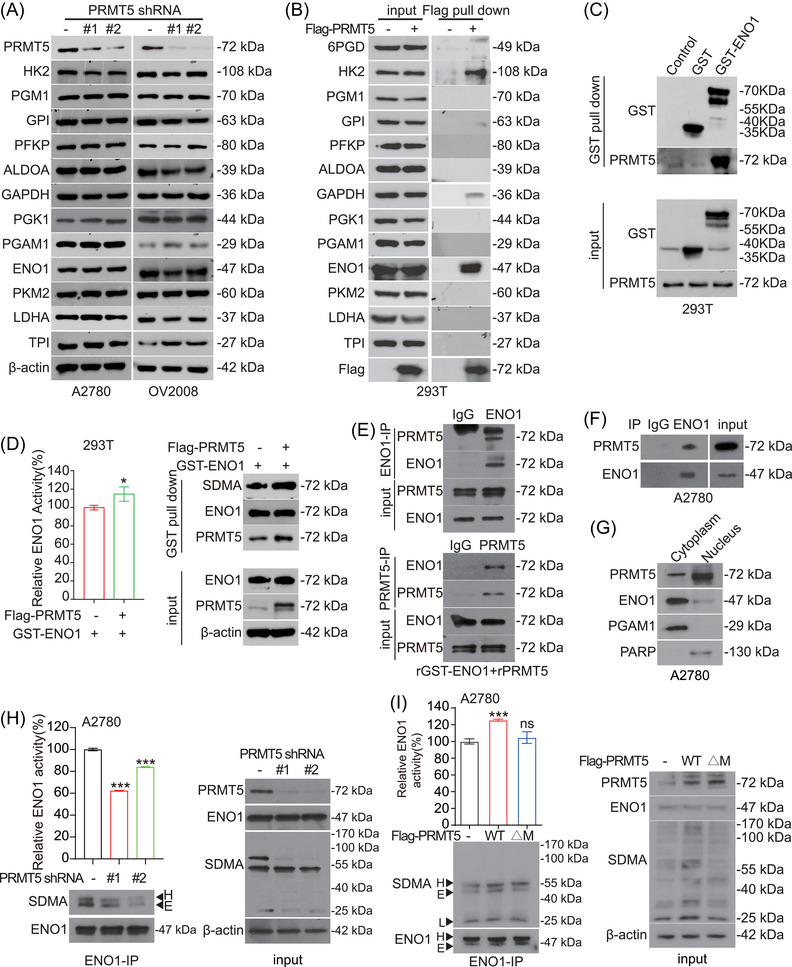
Targeting PRMT5 decreases ENO1 activity mediated by arginine methylation. (A) The protein expression of glycolytic enzymes was detected by western blotting in PRMT5 knockdown A2780 and OV2008 cells. (B) The interaction between glycolytic enzymes and PRMT5 was analyzed by Flag pull‐down assay. (C) The interaction between GST‐ENO1 and endogenous PRMT5 was analyzed by GST pull‐down assays in 293T cells. (D) GST‐ENO1 were pulled down from 293T cells that coexpressed Flag‐tagged PRMT5 or control vector, followed by ENO1 enzyme activity assay (*upper*) and western blotting (*lower*). (E) Recombinant PRMT5 and GST‐tagged ENO1 (rPRMT5 and rGST‐ENO1) purified from bacteria were incubated with each other in vitro. The interaction between ENO1 and PRMT5 was analyzed by Co‐IP. (F) A2780 extracts were immunoprecipitated with anti‐ENO1 or control rabbit IgG and western blotted by PRMT5 antibody. (G) Nucleocytoplasmic separation experiment was performed to detect PRMT5 and ENO1. (H) The endogenous enzymatic activity (*upper*) and SDMA level (*lower*) of ENO1 were analyzed in PRMT5 knockdown A2780 cells. (H, Heavy chain; E, Endogenous‐ENO1; L, Light chain.) (I) The endogenous enzymatic activity (*upper*) and SDMA level (*lower*) of ENO1 were analyzed in A2780 cells overexpressed PRMT5 WT or ΔM. (All error bars, mean values  ±  SD, p values were determined by unpaired two‐tailed Student's t test of n  =  3 independent biological experiments. * p < 0.05; **p < 0.01; ***p < 0.001).

To determine how PRMT5 impacts glycolysis pathway in ovarian cancer cells, we examined the effect of PRMT5 on metabolic enzymes involved in glycolysis pathway. First, we found that the expression levels of glycolytic enzymes were not changed in PRMT5 knockdown ovarian cancer cells compared with the control group (Figure 3A). So, we wondered whether PRMT5 regulates glycolysis through the interaction between PRMT5 and glycolytic enzymes. We performed Flag pull‐down assay to seek the metabolic enzymes of glycolysis pathway that could be coprecipitated with Flag‐tagged PRMT5 in HEK293T cells and found that hexokinase 2 (HK2), ENO1, and glyceraldehyde‐3‐phosphate dehydrogenase (GAPDH) were interacted with PRMT5 (Figure 3B). To validate the interaction between PRMT5 and HK2, ENO1, and GAPDH, we next performed GST pull‐down or Flag pull‐down to pull down endogenous PRMT5 using GST‐tagged ENO1, Flag‐tagged HK2, or Flag‐tagged GAPDH. We found that endogenous PRMT5 was also interacted with the exogenous expression of ENO1, HK2, and GAPDH (Figure 3C and Figure S2A–S2B). While the activity and SDMA level of exogenous ENO1, but not HK2 and GAPDH, were increased in HEK293T cells with exogenous expression of PRMT5 (Figure 3D and Figure S2C–S2D). Thus, we focused on the regulation of arginine methylation of ENO1 by PRMT5. in vitro Co‐IP assay demonstrated that purified recombinant PRMT5 and recombinant GST‐tagged ENO1 directly interact with each other (Figure 3E). In vivo co‐immunoprecipitation (Co‐IP) assay further demonstrated that endogenous ENO1 could bind to endogenous PRMT5 (Figure 3F). These results suggested that PRMT5 could interact directly with ENO1. In addition, the results of nucleocytoplasmic separation experiment showed that PRMT5 was distributed ubiquitously in both the nucleus and cytoplasm of A2780 cells, while ENO1 predominately showed cytoplasmic localization (Figure 3G). Lastly, we found that targeting PRMT5 by shRNA reduces the activity and SDMA level of ENO1 (Figure 3H). Meanwhile, overexpression of PRMT5 WT, but not inactivating mutant of PRMT5 (ΔM) promoted enzymatic activity and SDMA levels of ENO1 in A2780 cells (Figure 3I). Collectively, these data indicated that PRMT5 promotes ENO1 activity through regulating SDMA level on ENO1.

### 2.4 PRMT5 signals high glucose condition and increases ENO1 activity by methylating its R9 site

**FIGURE 4:**
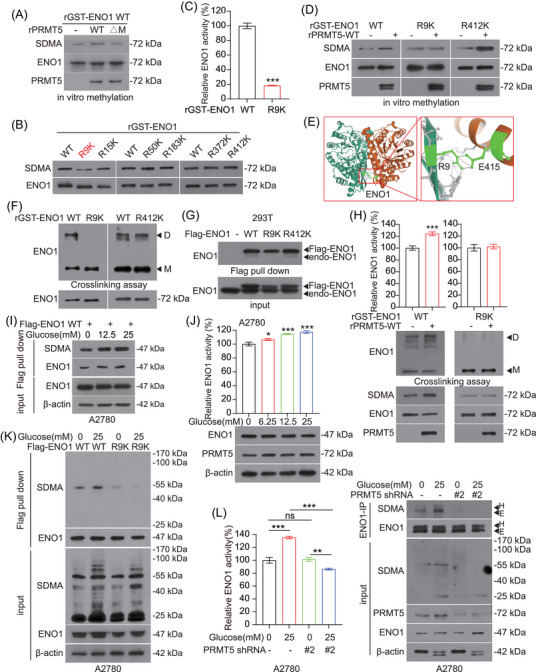
PRMT5 signals high glucose condition and to increase ENO1 activity by methylating its R9 site. (A) in vitro methylation assay was performed to detect the SDMA level of purified rGST‐ENO1, which was incubated with rPRMT5 wild type (WT) or inactivating mutant (ΔM). (B) The SDMA level of rGST‐ENO1 WT, R9K, R15K, R50K, R183K, R372K, and R412K were detected by western blotting. (C) The activity of purified rGST‐ENO1 WT and R9K was examined by ENO1 enzyme activity assay. (D) in vitro methylation assay was performed to detect the SDMA level of purified rGST‐ENO1, R9K, and R412K, which were incubated with or without rPRMT5 WT. (E) Structural location of R9 site on ENO1 was indicated (PDB ID 2PSN). (F) Crosslinking assay was performed to detect dimeric and monomeric ENO1 (*upper*; ENO1 antibody). ENO1 protein input assessed by western blotting is shown in the lower panel. (D, dimeric GST‐ENO1, 144 KD; M, monomeric GST‐ENO1, 72 KD). (G) Flag‐ENO1 WT, R9K, and R412K were expressed in 293T cells, followed by Flag pull‐down and western blotting to analyze the interaction between exogenous Flag‐ENO1 and endogenous ENO1. (H) Purified rGST‐ENO1 WT and R9K were incubated with or without rPRMT5 for in vitro methylation assay, followed by ENO1 enzymatic assays (*upper*), crosslinking assays (*middle*), and western blotting (*lower*). (I) The SDMA level of Flag‐ENO1 was detected by western blotting in A2780 cells, which treated with different concentrations of glucose for 12 h. (J) The activity and expression of ENO1 were detected by enzyme assay and western blotting in A2780 cells, which treated with different concentrations of glucose for 12 h. (K) The SDMA level of Flag‐ENO1 WT or ENO1 R9K was detected by western blotting in A2780 cells, which treated with or without 25 mM glucose for 12 h. (L) The activity (*left*) and SDMA level (*right*) of ENO1 were detected by enzyme assay and western blotting in PRMT5 knockdown or vector control A2780 cells, which treated with or without 25 mM glucose for 12 h. (I–L Before 0 or 25 mM sugar treatment, cells in each group were first subjected to glucose starvation for 6 h, and then normal cultured for 2 h. H, heavy chain; E, endogenous‐ENO1. All error bars, mean values  ±  SD, p values were determined by unpaired two‐tailed Student's t test of n  =  3 independent biological experiments. *p < 0.05; **p < 0.01; ***p < 0.001).

Next, we want to explore the underlying mechanism of PRMT5 in regulating ENO1 activity through methylating ENO1 directly. First, we incubated the recombinant GST‐tagged ENO1 purified from bacteria with Flag‐PRTM5, which pulled down from HEK239T cells, and we found that Flag‐PRMT5 WT, but not inactivating mutant (ΔM), methylated the recombinant GST‐tagged ENO1 (Figure S3A). In addition, we performed in vitro methylation assay with bacterially expressed PRMT5 and ENO1 proteins, and found that the SDMA level of ENO1 was increased by incubating with recombinant PRMT5 WT, but not inactivating mutant (ΔM) (Figure 4A). These results suggested that PRMT5 methylates ENO1 directly in vitro.

Proteomics analysis (PhosphoSitePlus powered by Cell Signaling Technology) revealed that there was a group of arginine methylation residues on ENO1 protein (https://www.phosphosite.org/proteinAction.action?id = 2610&showAllSites = true and https://www.phosphosite.org/siteTableNewAction?id = 2610&showAllSites = true).^33^ To examine the effect of arginine methylation on ENO1 activity, we constructed a series of ENO1 mutants in which arginine (R) was replaced with lysine (K). We found that substitution of arginine with lysine only at R9 site resulted in decreasing SDMA level of purified recombinant‐ENO1 compared to WT (Figure 4B). Meanwhile, the activity of ENO1 was also greatly reduced after mutating its R9 site (Figure 4C). Moreover, the R9 site of ENO1 was highly conserved among different species (Figure S3B). Based on the above findings, we performed protein and protein docking between the peptide consisting of six aa (HAREIF) adjacent to the R9 site of ENO1 and the active center of PRMT5 by Schrödinger, and found that R9 could interact with E435, which was identified as the catalytic site of PRMT5^34^ (Figure S3C). After further incubation with recombinant PRMT5 WT purified from bacteria (Figure 4D) or Flag‐PRMT5 WT pulled down from HEK293T cells (Figure S3D), ENO1 WT or R412K mutant showed an increased level of SDMA, whereas the R9K mutant showed a negligible change in SDMA level. Interestingly, our structural analysis demonstrated that R9 site is located at the interface between the two subunits of the ENO1 dimer (PDB ID: 2PSN) (Figure 4E). Thus, we speculated that ENO1 activity may be promoted by R9 methylation through affecting the formation of homodimers. To test this hypothesis, crosslinking experiments were performed and showed that the substitution of R9 residue by lysine residue could reduce the formation of ENO1 homodimer (Figure 4F). In addition, we also determined the interaction between Flag‐tagged ENO1 and endogenous ENO1 in HEK293 cells. As expected, ENO1 R9K mutant, but not WT or R412K mutant, disrupted the binding between Flag‐tagged ENO1 and endogenous ENO1 (Figure 4G). To further explore whether R9 methylation promotes ENO1 homo‐dimer formation is mediated by PRMT5, we then performed cross‐linking experiments after ENO1 was methylated by recombinant PRMT5 or Flag‐PRMT5. The results showed that PRMT5 promotes the homodimer formation and enzymatic activity of ENO1 WT, but not ENO1 R9K (Figure 4H and S3E). Furthermore, we found that knockdown of PRMT5 decreased the binding between Flag‐tagged ENO1 WT and endogenous ENO1, but did not affect the binding between Flag‐tagged ENO1 R9K and endogenous ENO1 (Figure S3F). Thus, these results revealed that PRMT5 promotes homodimer formation of ENO1 through methylates ENO1 at R9 site and enhances its activity.

We next explored the physiological condition for PRMT5 methylates R9 site of ENO1. As described in the Warburg effect, cancer cells increase glucose uptake and promote aerobic glycolysis, which are necessary for cell proliferation and rapid tumor growth.^35^ Therefore, we want to explore whether PRMT5 signals glucose to induce ENO1 methylation. Interestingly, the activity and methylation of ENO1 were increased under the increasing concentration of glucose stimulation (Figure 4I and J), but there is no significant change in the methylation level of Flag‐ENO1 R9K mutant (Figure 4K). When PRMT5 was blocked with shRNA followed by glucose stimulation, we no longer observed the increase in the methylation level and enzyme activity of endogenous ENO1 (Figure 4L and Figure S3G). These observations indicated that PRMT5 signals the glucose condition and methylates ENO1, which contributes to activate ENO1 enzymatic activity and glucose metabolism in ovarian cancer.

### 2.5 PRMT5 promotes cancer cell growth and glycolysis flux through activating ENO1

**FIGURE 5:**
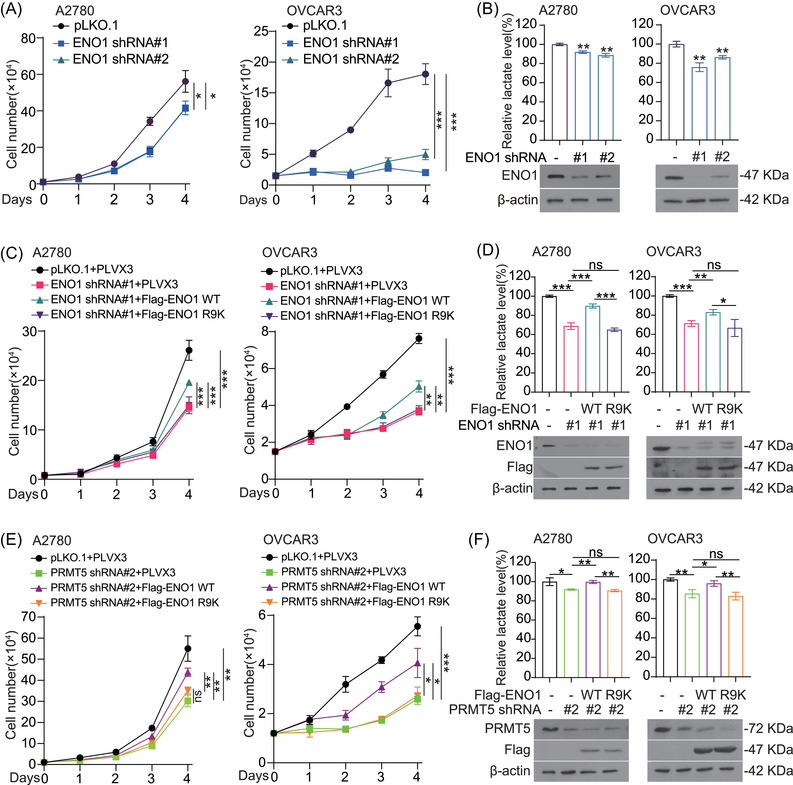
PRMT5 promotes cancer cell growth and glycolysis flux through activating ENO1. (A) Cell proliferation was determined by cell number counting in A2780 and OVCAR3 cells with ENO1 knockdown. (B) Relative lactate level (*upper*) was determined by Llactic Acid assay kit in A2780 and OVCAR3 cells with ENO1 knockdown. (C) Cell proliferation was determined by cell number counting in A2780 and OVCAR3 cells with ENO1 knockdown, which exogenous express Flag‐ENO1 WT or R9K (empty vector worked as a control). (D) Relative lactate level (*upper*) was determined by Lactic Acid assay kit in A2780 and OVCAR3 cells with ENO1 knockdown, which exogenous express Flag‐ENO1 WT or R9K (empty vector worked as a control). (E) Cell proliferation was determined by cell number counting in A2780 and OVCAR3 cells with PRMT5 knockdown, which exogenously express Flag‐ENO1 WT or R9K (empty vector worked as a control). (F) Relative lactate level (*upper*) was determined by Lactic Acid assay kit in A2780 and OVCAR3 cells with PRMT5 knockdown, which exogenously express Flag‐ENO1 WT or R9K (empty vector worked as a control). (All error bars, mean values  ±  SD, p values were determined by unpaired two‐tailed Student's t test of n  =  3 independent biological experiments. *p < 0.05; **p < 0.01; ***p < 0.001).

Since ENO1 is a major enzyme in glycolysis pathway and its activity and methylation were affected by PRMT5; thus, we tested whether PRMT5 promotes glycolysis pathway and cell proliferation by regulating ENO1. To test this hypothesis, we first knocked down ENO1 in ovarian cancer cells with shRNA and found that the depletion of ENO1 inhibits cell proliferation and reduces lactate production in ovarian cancer cells (Figure 5A and B and Figure S4A–S4B). This effect was partially rescued by exogenous expressed ENO1 WT not R9K mutant in the ENO1 knockdown cells (Figure 5C and D). Furthermore, the PRMT5 knockdown cells with exogenous expressed ENO1 WT not ENO1 R9K partially reversed the decreased cell proliferation (Figure 5E). A similar phenomenon was also observed in lactate production (Figure 5F and Figure S4C–S4D). Taken together, these data suggested that PRMT5 promotes cell proliferation and glycolysis flux via regulating activity of ENO1 through R9 methylation levels.

### 2.6 Targeting PRMT5 reduces ENO1 enzymatic activity, decreases glycolytic flux, and blocks ovarian cancer growth

**FIGURE 6:**
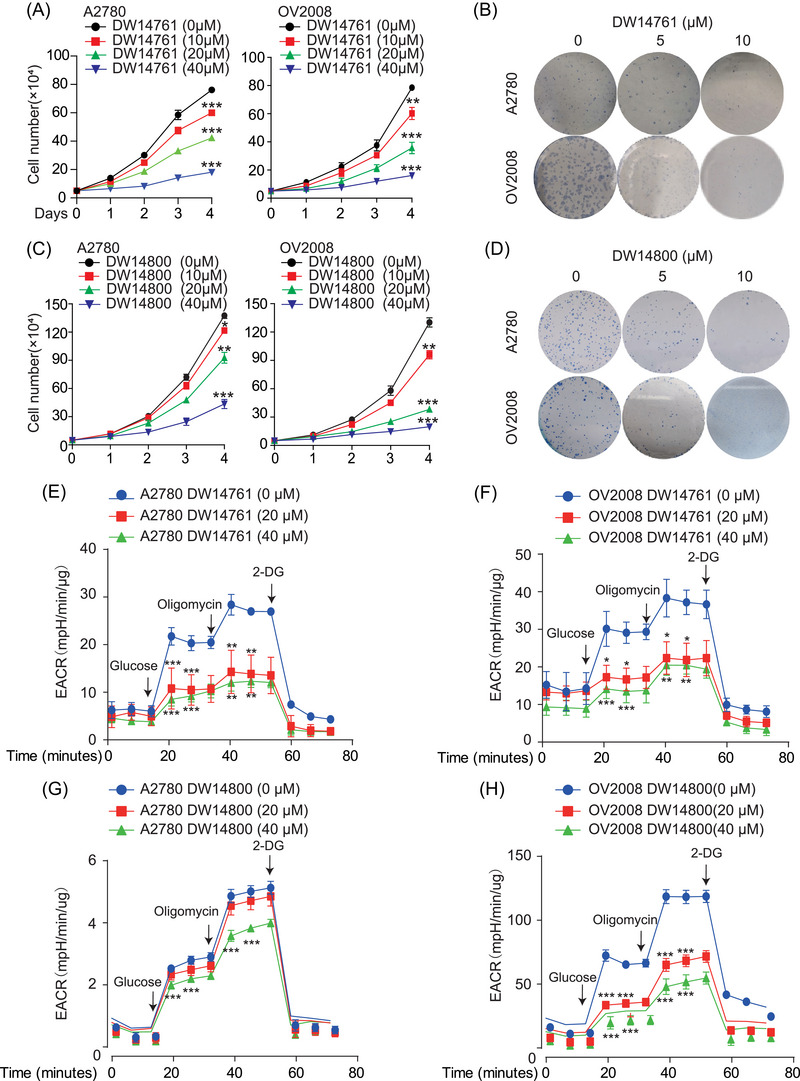
PRMT5 inhibitors reduce ENO1 enzymatic activity and glycolytic pathway. (A) The cell proliferation was determined by cell number counting in ovarian cancer cells treated with different concentrations of DW14761. (B) Colony formation assay was determined in ovarian cancer cells treated with different concentrations of DW14761. (C) The cell proliferation was determined by cell number counting in ovarian cancer cells treated with different concentrations of DW14800. (D) Colony formation assay was determined in ovarian cancer cells treated with different concentrations of DW14800. (E) The ECAR was measured in A2780 cells treated with different concentrations of DW14761. (2‐DG, 2‐Deoxy‐D‐glucose, the inhibitor of glycolytic pathway). (F) The ECAR was measured in OV2008 cells treated with different concentrations of DW14761. (G) The ECAR was measured in A2780 cells treated with different concentrations of DW14800. (H) The ECAR was measured in OV2008 cells treated with different concentrations of DW14800. (All error bars, mean values  ±  SD, p values were determined by unpaired two‐tailed Student's t test of n  =  3 independent biological experiments. *p < 0.05; **p < 0.01; ***p < 0.001).

In our previous study, DW14761 and DW14800 were efficient PRMT5 inhibitors designed and synthesized based on structural strategy.^36^ Thus, ovarian cancer cells were treated with different concentrations of DW14761 or DW14800 to evaluate the inhibitory effect of the two molecules on ovarian cancer by targeting PRMT5. Cell proliferation and colony formation assays showed that DW14761 (Figure 6A and B and Figure S5A–S5D) and DW14800 (Figure 6C and D and Figure S5E–S5H) exerted strong inhibitory effect on ovarian cancer cells in a time‐ and dose‐dependent manner. Furthermore, the glycolytic pathway was also inhibited by these two small molecules in ovarian cancer cells (Figure 6E–H and Figure S6A–S6D).

**FIGURE 7:**
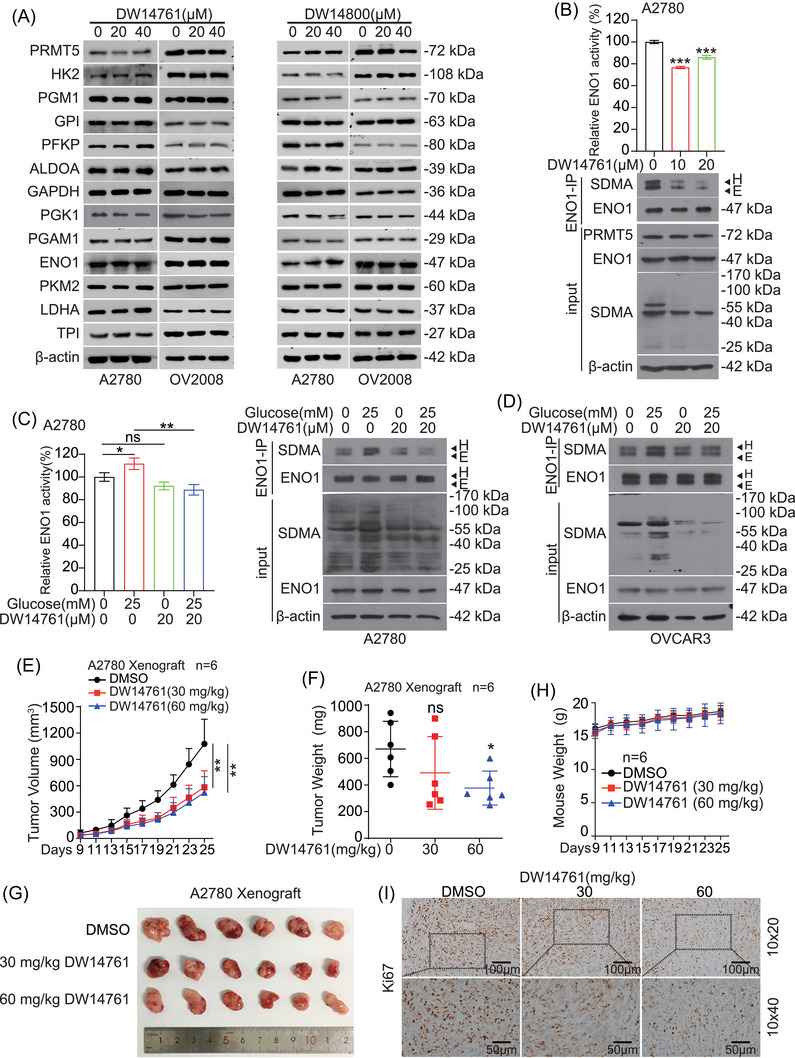
DW14761 as a PRMT5 inhibitor inhibits SDMA level and enzymatic activity of ENO1 and ovarian cancer growth. (A) The protein expression of glycolytic enzymes was detected by western blotting in ovarian cancer cells treated with different concentrations of DW14761 and DW14800. (B) The endogenous enzymatic activity (upper) and SDMA level (lower) of ENO1 were analyzed in A2780 cells treated with different concentrations of DW14761. (H, Heavy chain; E, Endogenous‐ENO1). (C) The activity (*left*) and SDMA level (*right*) of ENO1 were detected by enzyme assay and western blotting in A2780 cells, which treated with or without 20 μM DW14761 for 36 h, then followed by treatment with or without 25 mM glucose for 12 h. (H, Heavy chain; E, Endogenous‐ENO1). (D) The SDMA level of ENO1 was detected by western blotting in OVCAR3 cells, which treated with or without 20 μM DW14761 for 36 h, and then followed by treatment with or without 25 mM glucose for 12 h. (E) Tumor growth in xenograft nude mice injected with A2780 cells was compared between the group of mice treated with DW14761 and the control group treated with vehicle control. (F) Tumor weights in xenograft nude mice injected with A2780 cells were compared between the group of mice treated with DW14761 and the control group treated with vehicle control. (G) All tumors in xenograft nude mouse were shown. (H) Mice body weights in each group were recorded. (I) IHC was performed to detect the Ki67 level in a representative tumor. (All error bars, mean values  ±  SD, p values were determined by unpaired two‐tailed Student's t test of n  =  3 independent biological experiments. * p < 0.05; **p < 0.01; ***p < 0.001).

Next, we examined the effects of DW14761 and DW14800 on the expression of metabolic enzymes in glycolysis pathway. Consistent with the results of knockdown of PRMT5 in ovarian cancer cells, the two molecules had no effect on the protein levels of these enzymes (Figure 7A). Considering the inhibitory effect of the two molecules on the glycolysis pathway in A2780 cells (Figure 6E and G), we selected DW14761 for our next experiments. Treatment with DW14761 reduced the activity and SDMA level of ENO1 (Figure 7B) and prevented the increase in ENO1 activity and SDMA level in response to glucose (Figure 7C and D). Furthermore, we tested antitumor effects of DW14761 In vivo using A2780 cells CDX mouse model. DW14761 was administrated intraperitoneally (ip) with doses of 30 and 60 mg/kg every 2 days into xenograft mice. We found that DW14761 significantly reduced tumor growth and tumor masses in DW14761 treated mice compared to mice receiving DMSO (Figure 7E–G), without affecting mice weight (Figure 7H). Meanwhile, the Ki67 (cell proliferation markers, proliferating cell nuclear antigen) expression was decreased in DW14761‐treated tumor tissues (Figure 1I). Taken together, these data suggested that DW14761 significantly suppresses ovarian cancer growth In vivo.

### 2.7 Targeting PRMT5 enhances antitumor effect of Taxol in ovarian cancer

**FIGURE 8:**
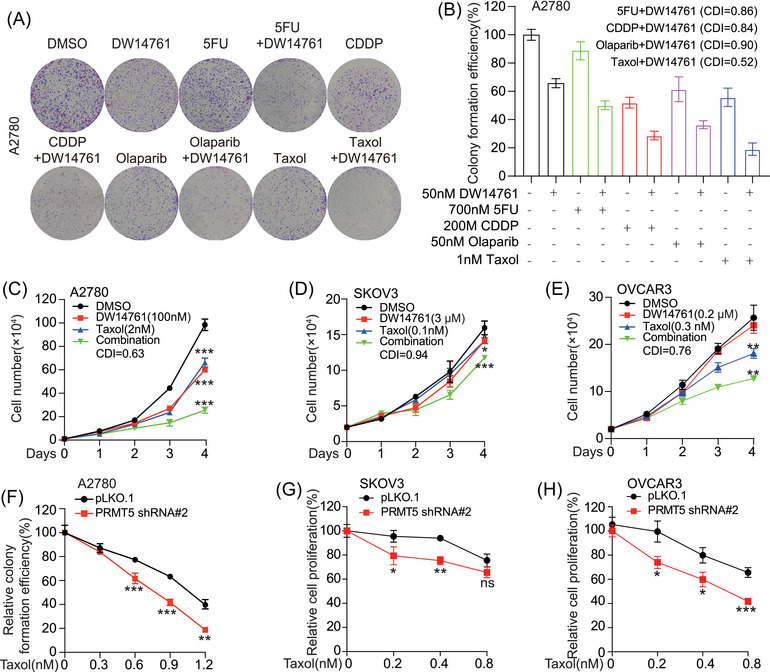
Targeting PRMT5 enhances antitumor effect of Taxol in ovarian cancer. (A) Colony formation was determined in A2780 cells treated with DW14761 and chemotherapy drugs (including 5‐FU, Cisplatin, Olaparib, and Taxol), alone or in combination. (B) Statistical analyses of colony formation were performed. (C) Cell proliferation was determined by cell number counting in A2780 cells treated with DW14761 or Taxol, alone or in combination. (D) Cell proliferation was determined by cell number counting in SKOV3 cells treated with DW14761 or Taxol, alone or in combination. (E) Cell proliferation was determined by cell number counting in OVCAR3 cells treated with DW14761 or Taxol, alone or in combination. (F) Cell proliferation was determined by colony formation in A2780 cells with stable PRMT5 knockdown when treated with or without different concentrations of Taxol. (G) Cell proliferation was determined by cell number counting in PRMT5 knockdown or vector control SKOV3 cells treated with different concentrations of Taxol. (H) Cell proliferation was determined by cell number counting in PRMT5 knockdown or vector control OVCAR3 cells treated with different concentrations of Taxol. (All error bars, mean values  ±  SD, p values were determined by unpaired two‐tailed Student's t test of n  =  3 independent biological experiments. *p < 0.05; **p < 0.01; ***p < 0.001).

To further determine the therapeutic benefit of targeting PRMT5 in combination with chemotherapy drugs, we tested the antitumor effect of chemotherapy drugs, including 5‐FU, Cisplatin, Olaparib, and Taxol by combination targeting with PRMT5. Through colony formation assay, we found that the treatment of DW14761 significantly enhanced the antitumor effect of these chemotherapy drugs, of which Taxol had the best effect (Figure 8A and B). Furthermore, the combination of DW14761 and Taxol markedly inhibited the cell proliferation, compared to either DW14761 or Taxol treatment alone (Figure 8C–E). Meanwhile, knockdown of endogenous PRMT5 also robustly enhanced the sensitivity of ovarian cancer cells to Taxol treatment (Figure 8F–H and S7A). Taken together, these data suggested that the antitumor effects of Taxol in ovarian cancer were significantly enhanced by targeting PRMT5.

## 3 DISCUSSION

PRMT5 is a major Type‐II arginine methyltransferase that catalyzes SDMA on protein arginine residues.^11^ Aberrant expression of PRMT5 has been reported to be associated with various human cancers,^10^, ^37^, ^38^ including epithelial ovarian cancer.^39^ Interestingly, we also found that PRMT5 was highly expression in ovarian cancer tissues than normal ovarian tissues based on public databases and our data cohort. Furthermore, the high expression of PRMT5 is associated with poor prognosis in patients with ovarian cancer. Therefore, our results suggested that PRMT5 may be a potential therapeutic target in ovarian cancer. Indeed, suppressing PRMT5 using shRNA or inhibitors (DW14761 and DW1480) significantly reduced ovarian cancer colony formation, cell proliferation, and tumor growth. Lastly, we found that PRMT5 was also increased in various human ovarian cancer cells. Notably, although the protein level of PRMT5 was significantly increased in ovarian cancer cells, the increase at the mRNA level was relatively weak (Figure 1D). It has been reported that PRMT5 is highly expressed in ovarian cancer, but no mRNA has been detected.^39^ This reminds us that posttranscriptional regulation may be an important mechanism for the high expression of PRMT5 in ovarian cancer and deserves further exploration. In addition, among our ovarian cancer cells, A2780‐Taxol and OVCAR3‐Taxol are Taxol‐resistant A2780 and OVCAR3 cells, from the parental A2780 and OVCAR3 cells, by exposure to increasing concentrations of Taxol.^40^ Interestingly, we observed that PRMT5 expression was higher in A2780‐Taxol (C13*, OVCAR3‐Taxol) than that in parental A2780 (OV2008, OVCAR3). Taxol is a chemotherapeutic drug clinically used in the treatment of ovarian cancer, and it acts in a complex manner to alter a variety of cellular carcinogenesis processes, such as angiogenesis, apoptosis, mitosis and ROS production, and so on. However, chemoresistance to Taxol during clinical treatment is a major obstacle limiting the efficacy of antitumor chemotherapy. Mechanistically, many cellular processes, including autophagy, oxidative stress, epigenetic alterations, and microRNA deregulation, contribute to Taxol resistance in cancer cells.^41^ Combined with the above observations in our manuscript, we speculate that, on the one hand, high levels of PRMT5 may serve as a biomarker for Taxol resistance in ovarian cancer, and, on the other hand, high expression of PRMT5 may be an important mechanism of cancer cell resistance to Taxol. The molecular mechanism deserves further exploration. Moreover, we found that targeting PRMT5 could enhance antitumor effect of Taxol in ovarian cancer. However, how inhibition of PRMT5 enhances antitumor response of Taxol in ovarian cancer was not explored in this study.

An expanding literature demonstrates the role of PRMT5 in regulating a wide range of cellular processes, by methylating histone and nonhistone proteins methylation.^42^ For example, high PRMT5 expression drives transcriptional silencing of tumor‐suppressing miR33b, miR96, and miR503 via histone 3 dimethylation in their promoter regions, and then reinforces lymphoma cell growth and survival.^43^ Nagai et al. reported that forkhead box protein P3 (Foxp3) can be dimethylated at positions R27, R51, and R146 by PRMT5 and enhances Treg function.^44^ Bao et al. found that PRMT5 regulates epithelial ovarian cancer cell growth and apoptosis via modulating the expression of E2F transcription Factor 1 (E2F‐1).^39^ The cell metabolism reprogramming has been considered as a hallmark of cancer, including ovarian cancer. Qin et al. reported that PRMT5 enhances glycolysis in pancreatic cancer via the F‐box/WD repeat‐containing protein 7 (FBW7)/cMyc axis.^45^ Han et al. findings show that PRMT5 promotes aerobic glycolysis of breast cancer cells by regulating the liver X receptor α (LXRα)/NF‐κBp65 pathway.^46^ However, the roles and mechanisms of PRMT5 in controlling ovarian cancer progression have not yet to be fully determined. In this study, we revealed a crucial role of PRMT5 in promoting ovarian cancer by regulating glycolysis flux. In addition, we demonstrated that PRMT5 is a critical enzyme for ENO1 arginine symmetric dimethylation modification and enhance its activity, which regulates glycolysis pathway, and promotes ovarian cancer growth. Thus, we first reported that PRMT5 enhances glycolysis in ovarian cancer by interacting with and methylating ENO1.

Next, we explored the mechanism of PRMT5‐mediated arginine symmetric dimethylation on ENO1. Several studies have proved that PTMs, such as phosphorylation and acetylation, affect the occurrence and development of various cancer by regulating metabolic enzymes and glycolysis.^47^ According to previous reports, ENO1 is regulated by various PTMs, affecting its enzymatic activity in the glycolytic pathway.^27^, ^28^, ^29^, ^30^ In particular, a previous work discovered that PRMT5 monomethylates ENO1 at R50 to regulate its LPS‐induced translocation from the cytoplasm to the cell surface of lung epithelial cancer cells, thereby affecting the invasive properties of cancer cells.^48^ Interestingly, our study indicated that ENO1 is also a symmetric arginine dimethylated protein modified by PRMT5, and demonstrated that PRMT5 could directly methylate ENO1 in vitro. Moreover, we further revealed that symmetrical dimethylation at R9 of ENO1 promotes the formation of ENO1 dimers, which increases ENO1 enzymatic activity and enhances glycolysis. Lastly, we found that the high methylation level and activity of ENO1 mediated by PRMT5 were stimulated by high concentration of glucose. The results in Figure 4I indicated that glucose did not affect the protein level of PRMT5. Previous reports have shown that glucose‐derived ribose and one‐carbon units provided by glucose and serine metabolism are cooperatively integrated into the methionine cycle through de novo ATP synthesis and promote S‐adenosylmethionine (SAM) generation.^49^ Therefore, we speculate that high concentration of glucose may promote intracellular methylation level by increasing the content of SAM. In addition, there is also a review summarizing that there are many different PTMs in different structural domains of PRMT5, especially phosphorylation, which regulate the function of PRMT5 through different mechanisms.^50^ It is well known that the glucose also has an important impact on the PTM in cells, so we speculate that glucose may also affect the function of PRMT5 by regulating the PTM of PRMT5. In conclusion, the effect of glucose on the methylation level regulated by PRMT5 deserves more research to be explored. In addition, we also observed that PRMT5 interacts with HK2 and GAPDH in the glycolytic pathway. But, here, we do not explore its biological functions in ovarian cancer. Thus, the biological functions of PRMT5 binding to HK2 or GAPDH deserves further exploration, which would hopefully help us to broader implications for proving PRMT5 as a potential target for the treatment of ovarian cancer.

Interestingly, in our previous report, we demonstrated that PRMT6 asymmetrically dimethylates the R9 and R372 sites of ENO1 in lung cancer,^51^ which is different from our finding in this study that PRMT5 only symmetrically dimethylates the R9 site of ENO1 in ovarian cancer. Although PRMT5 and PRMT6 are not the same type of PRMTs, there is a certain degree of similarity in the selection of substrate catalytic sites. Both of them could methylate the R9 site of ENO1. This situation is similar to that summarized in a previous review. H4R3 can be catalyzed by various methyltransferases for different methylation modifications under different circumstances.^52^ Our results first revealed that nonhistone arginine residues are also methylated by different methyltransferases. At the same time, we raise a bold question based on these findings whether symmetric and asymmetric dimethylation co‐occur on the same arginine residue. Although we did not simultaneously explore the mechanisms of PRMT5 and PRMT6 on ENO1 in two cancer types, our findings did make us feel that the above question deserves further exploration. Of course, it is also very likely that different methyltransferases regulate the same arginine residue through different mechanisms in different cancer types. In addition, we found that the expression of PRMT5 was higher in lung cancer than in normal lung tissue. However, knockdown of PRMT5 promoted the proliferation of lung cancer cells.^51^ In another report,^40^ we found that the mRNA of PRMT6 in paclitaxel‐resistant ovarian cancer cells was lower than that in control ovarian cancer cells (Figure 6A and B). In this manuscript, PRMT5 is highly expressed in ovarian cancer cells, and the expression level is higher in paclitaxel‐resistant cells. Meanwhile, PRMT5 promotes the proliferation of ovarian cancer (Figure 1). Taking together, PRMT5 and PRMT6 may regulate tumor progression through different mechanisms in ovarian cancer and lung cancer, respectively. It is worthy to carry out more experimental research to explore the potential mechanisms.

There are several PRMT5 inhibitors have been developed by different pharmaceutical companies, and some of these compounds have entered clinical trials. The fastest progress is GSK3326595 from GlaxoSmithKline, which is currently in phase II clinical studies.^5^ However, these compounds still have a lot limited use in treating cancer. Thus, it is urgent to develop new inhibitor to overcome these limited use. In our previous study, DW14761 and DW1480 were designed with structure‐based approach and synthesized as a highly potent inhibitor of PRMT5 based on structure, and DW14761 has a high specificity against PRMT5 relative to other PRMTs. Our previous results showed that DW14761 significantly decreased the level of SDMA in leukemia MV4‐11 cells and inhibited tumor growth in MV4‐11 cells xenograft mouse model.^36^ However, the function of DW14761 by targeting PRMT5 in ovarian cancer has not been explored. In the present study, we found that targeting PRMT5 activity by using DW14761 significantly suppressed ovarian cancer growth by regulating glycolysis through methylating ENO1.

Taxol (Paclitaxel) is a natural anticancer drug, which exerts antitumor effect by inhibiting mitosis and has been widely used in clinical treatment of ovarian cancer.^41^, ^53^ However, there are several different mechanisms to induce resistance of the cancer cells to Taxol, thereby reducing its clinical therapeutic effect.^54^ Prior to this, some researchers have begun to evaluate the synergistic effect of Taxol and other targeted therapeutic agents in adjuvant and neoadjuvant treatment programs for ovarian cancer.^55^ In this study, we explored whether targeting PRMT5 could increase the sensitivity of ovarian cancer to Taxol treatment. Indeed, the knockdown of endogenous PRMT5 significantly enhanced the antitumor effects of Taxol on ovarian cancer. As expected, DW14761 treatment also increased the sensitivity of ovarian cancer to Taxol. Thus, our results provided a new perspective for the combined use of Taxol and other therapeutics in the treatment of ovarian cancer.

In this study, we identified PRMT5 as a driver for ovarian cancer in promoting ovarian cancer progression. Mechanistically, we revealed that PRMT5 enhances glycolytic flux through regulating the ENO1 activity mediated by SDMA, which is critically required for the formation of active ENO1 dimer. In summary, our study suggested the mechanisms and roles of PRMT5 in promoting ovarian cancer growth, and targeting PMRT5 could be provided new potential clues for the treatment of ovarian cancer.

## 4 MATERIALS AND METHODS

### 4.1 Biological resources and reagents

The Biological Resources and Reagents are listed in KEY RESOURCES TABLE of Table S3.

### 4.2 Cell culture

HEK293T, HosEpic, A2780, and A2780‐Taxol cells were obtained from Dr. Zhi Shi (Jinan University, Guangdong, China). IOSE‐80, Hey‐T30, and SKOV3 cells were purchased from Procella (Wu Han, China). HEK293T, OVCAR3, OVCAR3‐Taxol, and HosEpic cells were cultured in Dulbecco's modified Eagle's medium (DMEM, Thermo Fisher Scientific, MA, USA) supplemented with 10% fetal bovine serum (FBS, ExCell Bio, Shanghai, China). IOSE‐80, A2780, A2780‐Taxol, Hey‐T30, and SKOV3 cells were maintained in RPMI‐1640 medium (Thermo Fisher Scientific, MA, USA) supplemented with 10% FBS. Human ovarian surface epithelial cancer cells OV2008 and C13* were gifts from Dr. Benjamin K. Tsang (University of Ottawa, Ontario, Canada). OV2008 and C13* cells were cultured in RPMI 1640 medium with 10% FBS. Cells were cultured at 37°C in an atmosphere of 5% CO2/95% air (normoxic conditions) at 37°C. For glucose‐starved culture, cells were washed with phosphate buffer saline (PBS) and then incubated with glucose‐free RPMI‐1640 medium containing 1% FBS. For the cells that need to be treated with drugs, the cells were treated with the indicated concentration of drugs for corresponding time (refer to the corresponding figure legends for drug concentration and time scale of the experiment) after cell attachment, and then the subsequent experiments were carried out.

### 4.3 Clinical material

Thirty nine clinical ovarian cancer tissues and 22 normal tissues (Cohort 1) were collected from Tianjin Central Hospital of Gynecology Obstetrics (Tianjin, China) after surgical resection. The ovarian cancer tissues (Cohort 2 = 65 ovarian cancer tissues) were purchased from Shanghai Outdo Biotechnology Co., Ltd. (Shanghai Outdo Biotechnology, Shanghai, China). The related features were shown in Table S3. The clinical ovarian cancer and normal specimens all have the written consent approving the use of the samples for research purposes from patients. The study protocol was approved by the Institute Research Ethics Committee at Nankai University and Tianjin Central Hospital of Gynecology Obstetrics.

### 4.4 Gene knockdown with shRNA or siRNA

Stable knockdown of endogenous PRMT5 or ENO1 was achieved using lentiviral vector harboring shRNA purchased from Transheep Biological Corporation (Transheep, Shanghai, China). To generate cells with stable knockdown of PRMT5 or ENO1, lentiviral vector and packing plasmids (pMD2.G and psPAX2) were first transfected into HEK293T cells, and the lentiviral particles filtered through 0.45‐μm filter were infected into A2780 cells, followed by puromycin selection of the infected cells.

siRNA targeted for PRMT5 were purchased from GenePharma (GenePharma, Shanghai, China) and were transfected into target cells using siRNA‐mate (GenePharma, Shanghai, China) according to the manufacturer's instructions.

### 4.5 Plasmid constructs and transfections

Human PRMT5 cDNA (NM_006109.5) and ENO1 cDNA (NM_001428.5) were cloned into pLVX3 with 3X Flag‐tag at N‐terminus or pETM3C vector with his tag. The PRMT5 ΔM and PRMT5 slient mutation (SM#2, against PRMT5 shRNA#2) were constructed in pLVX3 (or pETM3C) using the Fast Mutagenesis System (TRAN, Beijing, China). R9K, R15K, R50K, R183K, R372K, and R412K mutations were constructed in pLVX3 (or pETM3C) using the Fast Mutagenesis System (TRAN, Beijing, China) with GST tag at the N‐terminus of ENO1. The sequences of these primers used in these are given in Table S3. For transient transfections, cells were grown to 80% confluency and transfected with plasmids using polyethylenimine (PEI) Transfection Reagent (Polysciences, IL, USA) according to the manufacturer's protocol.

### 4.6 Seahorse assay

We use an XF‐96 Extracellular Flux Analyzer (Seahorse Bioscience, Agilent Technologies, CA, USA) to analyze the real‐time bioenergy changes of the ECAR as described previously.^56^ In brief, the cells infected with pLKO.1 or shRNA were cultured in the corresponding medium as described in “4.2 cell culture.” If the cells need to be treated with drugs, the corresponding concentration of drugs was added for 48 h after the cells adhered to the wall. For analyzing real‐time bioenergy changes in ECAR, we plated cells into XF‐96 cell culture plates in “Seahorse medium,” and 1 × 104 cells were inoculated in each well. Then the stimuli were injected into the wells by the XF analyzer. The data were plotted as ECAR (mpH/min/μg) as a function of time after normalizing to cell numbers.

### 4.7 Lactate production assays

For lactate production assays, cells were seeded into six well plates and incubated for 24 h at 37°C. After aspirating the original medium from each well, cells were washed with PBS, and then incubated with phenol red‐free RPMI 1640 medium for 1 h. Lactate production in the medium was detected by the Lactic Acid assay kit (Nanjing Jiancheng Bioengineering Institute, Nanjing, China) according to the manufacturer's instruction. Relative lactate levels were normalized to cell numbers by counting.

### 4.8 ENO1 activity assay

ENO1 activity was measured in the reaction buffer containing 50 mmol/L Tris‐HCl (PH 7.5), 100 mmol/L KCl, 25 mmol/L MgSO4, 1.3 mmol/L ADP (Sigma, MO, USA), and 0.15 mmol/L nicotinamide adenine dinucleotide (NADH, Sigma, MO, USA). In addition, 0.6–1 U of pyruvate kinase and 0.9–1.4 U of lactate dehydrogenase (Sigma, MO, USA) were added to the reaction solution. After adding 1.9 mmol/L 2‐phosphoglycerate (2‐PG, Shanghai yuanye Bio‐Technology, Shanghai, China) and appropriate amount of ENO1 (1 μg of purified rENO1 or 10 μg of cell lysates), the absorbance in 340 nm as a measure of NADH was recorded at room temperature every 1 min for 10−30 min on a Microplate Photometer (Thermo Fisher Scientific, MA, USA).

### 4.9 In vitro methylation assays

In vitro methylation reactions were performed in 50 μL reaction system with PRMT5, ENO1, and 16 μmol/L S‐adenosyl‐methionine (SAM, Sigma, MO, USA), and supplemented with TBS (50 mmol/L Tris, 150 mmol/L NaCl). The reactions were incubated at 30°C for 1.5 h and stopped by the addition of sample loading buffer for western blot (WB).

### 4.10 Crosslinking assay

After performing in vitro methylation reaction, purified recombinant ENO1 was incubated at room temperature for 5 min in the absence or presence of 0.0025% glutaraldehyde containing 5% glycerol, 20 mM Tris‐HCl (pH 7.5), 100 mmol/L NaCl, 0.2 mmol/L ethylene diamine tetraacetic acid, and 2 mmol/L dithiothreitol. Crosslink reaction should be terminated by adding SDS sample buffer, skip the boiling sample step, and followed by SDS‐PAGE. The same amounts of noncrosslinking proteins were loaded to ensure equivalent protein amounts.

### 4.11 RNA extraction and RT‐qPCR

Total RNA was isolated using TRIzol (15596018, Gibco) reagent. cDNA was synthesized using Hifari III 1st Strand cDNA Synthesis SuperMix for qPCR (11141ES60, YEASEN, Shanghai, China). Real‐time quantitative PCR (RT‐qPCR) was performed using Hieff qPCR SYBR Green Master Mix (No Rox) (11201ES08, YEASEN, Shanghai, China).

### 4.12 Flag pull‐down, GST pull‐down, and (Co‐) immunoprecipitation assay

NP40 lysis buffer with cells was incubated on ice for 20 min, and then centrifuged at 12,000 rpm for 10 min at 4°C. Flag (GST)‐conjugated beads or agarose preconjugated with the appropriate antibody for 2 h was added to the supernatant and incubated overnight at 4°C. Immunoprecipitates obtained by centrifugation were washed three times with prechilled PBS and subjected to western blotting using specific antibodies.

### 4.13 Immunohistochemical staining

For immunohistochemical (IHC) staining, sections obtained from formalin‐fixed paraffin‐embedded samples were dewaxed and rehydrated following standard methods. Antigen retrieval was performed by boiling samples in citrate buffer for 15 min and endogenous peroxidase was blunted by incubating samples with 3% hydrogen peroxidase for 10 min. Sections were blocked with 10% normal goat serum (NGS) (ZSGB‐BIO, Beijing, China) for 20 min at room temperature and then incubated overnight at 4°C with primary antibodies (Ki67) (Cell signaling technology, Boston, USA) diluted in PBST (0.03% Triton X‐100 in PBS) containing 10% NGS. After rinsing with PBST and incubating with secondary antibodies, all paraffin sections were developed with DAB (MXB Biotechnologies, Fuzhou, China) and counterstained with hematoxylin. Finally, the sections were dehydrated through an ethanol series, sealed with neutral resin and photographed under a microscope.

We used ImageJ for Immunohistochemical staining analysis, the methods refer to https://www.dentalearner.com/archives/2541.

### 4.14 Animals

Nude mice (BALB/C nude, Female, 4−6‐week‐old) were purchased from Charles River (Beijing, China). To establish A2780 cells CDX models, 5 × 10^6^ A2780 cells harboring empty vector were injected into mice. The mice were performed according to the institutional ethical guidelines for animal experiments. Two perpendicular diameters were measured to calculate tumor growth using equation 4π/3 × (length/2) × (width/2).^2^ Mice were sacrificed 22 days after inoculation, and the subcutaneous tumors were removed, weighed, and their sizes were measured.

To investigate the effects of DW14761 on tumorigenesis In vivo, mice with A2780 xenografts were injected ip with DW14761 (vehicle control, 30 mg/kg or 60 mg/kg) every 2 days, whereas tumor volume and mice weight were recorded. To explore the combination effect of DW14761 and Taxol on tumor growth of ovarian cancer, mice with A2780 xenografts were injected ip every 2 days with DW14761 (30 mg/kg), Taxol (10 mg/kg) (Meilunbio, Dalian, China), or in combination, and tumor volume and mice body weight were recorded during the treatment.

### 4.15 Bioinformatics analysis

We use the public datasets TCGA and GEO datasets to perform bioinformatics analysis. Kaplan–Meier plotter (http://kmplot.com/analysis/index.php?p = background) was used to analyze overall survival. In addition, Survival and Survminer packages (The R Foundation for Statistical Computing, Vienna, Austria) in R were used to analyze patient survival and prognosis.

### 4.16 Statistical analysis

Data were analyzed using GraphPad Prism 8 software. Statistical details of experiments include p values, error bars, and mean values ± SD. Unpaired two‐tailed Student's *t* test was used to analyze the significant difference between two groups. Asterisk means a p value < 0.05 and was considered statistically significant.

## AUTHOR CONTRIBUTIONS

Fei Xie: drafting of the manuscript; Fei Xie, Kongkai Zhu, Cheng‐Shi Jiang, Han Zhang, Hongkai Chang, Yaya Qiao, Xiaoya Zhang, Mingming Sun, Jiyan Wang, Junzhen Tan: acquisition of data, analysis and interpretation of data, statistical analysis; Kongkai Zhu, Cheng‐Shi Jiang, Shuangping Liu, Tao Wang, Lianmei Zhao, Yuan Zhang, Chunze Zhang, Jianguo Zhao, Cheng Luo: technical and/or material support; Mukuo Wang, Jianping Lin: performing protein–protein docking; Jianguo Zhao, Shuai Zhang, Changliang Shan: funding acquisition; Shuai Zhang, Changliang Shan: study concept and design, study supervision; Changliang Shan: writing‐reviewing and editing. All authors have read and approved the final manuscript

## CONFLICTS OF INTERESTS

The authors have declared that no competing interest exists.

## ETHICS APPROVAL

This study was carried out in accordance with the recommendations of Requirements of the Ethical Review System of Biomedical Research Involving Human by National Health and Family Planning Commission of China, Nankai University Laboratory Animal Welfare Ethics Review Committee (Approval No.2022‐SYDWLL‐000355), Biomedical Ethics Committee of Nankai University (Ethics Document NO.NKUIRB2022022), and Tianjin Central Hospital of Gynecology Obstetrics with written informed consent from all subjects. All subjects were given a written informed consent in accordance with the Declaration of Helsinki.

## FUNDING INFORMATION

This work was supported by grants from National Natural Science Foundation of China (81973356 to C.S and 82273963 to S.Z), the Natural Science Foundation of Tianjin (21JCZDJC00060 to C.S), supported by the Open Fund of Tianjin Key Laboratory of human development and reproductive regulation (2020XHY02 to C.S) and Tianjin Key Medical discipline (Specialty) Construction Project (TJYXZDXK‐043A), and the Fundamental Research Funds for the Central Universities of Nankai University.

## Supporting information

Supporting Information

## Data Availability

The data that support the findings of this study are available from the corresponding author upon reasonable request.
