# Supplementary material for: PRMT5 promotes ovarian cancer growth through enhancing Warburg effect by methylating ENO1
Source: MedComm (2020). 2023 Mar 28;4(2):e245. doi: 10.1002/mco2.245 (PMC10044308; doi:10.1002/mco2.245)
Supplement: Supplementary file 1 — Supporting Information [file MCO2-4-e245-s001.docx]

**Supplementary information**

***PRMT5 promotes ovarian cancer growth through enhancing Warburg Effect by methylating ENO1***

*Short title: PRMT5 promotes ovarian cancer growth*

Fei Xie^1,*^, Han Zhang^1,*^, Kongkai Zhu^2,*^, Cheng-Shi Jiang^3,*^, Xiaoya Zhang^4^, Hongkai Chang^1^, Yaya Qiao^1^, Mingming Sun^1^, Jiyan Wang^1^, Mukuo Wang^1^, Junzhen Tan^5^, Tao Wang^6^, Lianmei Zhao^7^, Yuan Zhang^8^, Jianping Lin^1^, Chunze Zhang^9^, Shuangping Liu^10^, Jianguo Zhao^6,#^, Cheng Luo^11,#^, Shuai Zhang^5,#^ , Changliang Shan^1,#^

^1^State Key Laboratory of Medicinal Chemical Biology, College of Pharmacy and Tianjin Key Laboratory of Molecular Drug Research, Nankai University, Tianjin 300350, China.

^2^Advanced Medical Research Institute, Shandong University, Jinan, 250014, China.

^3^School of Biological Science and Technology, University of Jinan, Jinan, 250022, China.

^4^Biomedical Translational Research Institute, Jinan University, Guangzhou, Guangdong 510632, China.

^5^School of Integrative Medicine, Tianjin University of Traditional Chinese Medicine, Tianjin 301617, China.

^6^Tianjin Key Laboratory of human development and reproductive regulation,Tianjin Central Hospital of Obstetrics and Gynecology, Tianjin 300100, China.

^7^Research Center, The Fourth Hospital of Hebei Medical University, Shijiazhuang, Hebei 050011, China.

^8^The Sixth Affiliated Hospital of Guangzhou Medical University, Qingyuan, Guangdong, 511518, China.

^9^Department of Colorectal Surgery, Tianjin Union Medical Center, Nankai University, Tianjin 300121, China.

^10^Department of Pathology, Medical School, Dalian University, Dalian, Liaoning 116622, China.

^11^State Key Laboratory of Drug Research, Shanghai Institute of Materia Medica, Chinese Academy of Sciences, Shanghai 201203, China.

* These authors contributed equally.

# Corresponding Author: E-mail: Changliang Shan - [changliangshan@nankai.edu.cn](mailto:changliangshan@nankai.edu.cn), Shuai Zhang - [shuaizhang@tjutcm.edu.cn](mailto:shuaizhang@tjutcm.edu.cn), Cheng Luo - [cluo@simm.ac.cn](mailto:cluo@simm.ac.cn), Jianguo Zhao - [killingyousoft@126.com](mailto:killingyousoft@126.com(J.Z),).

**Supplementary Figure Legends**

**
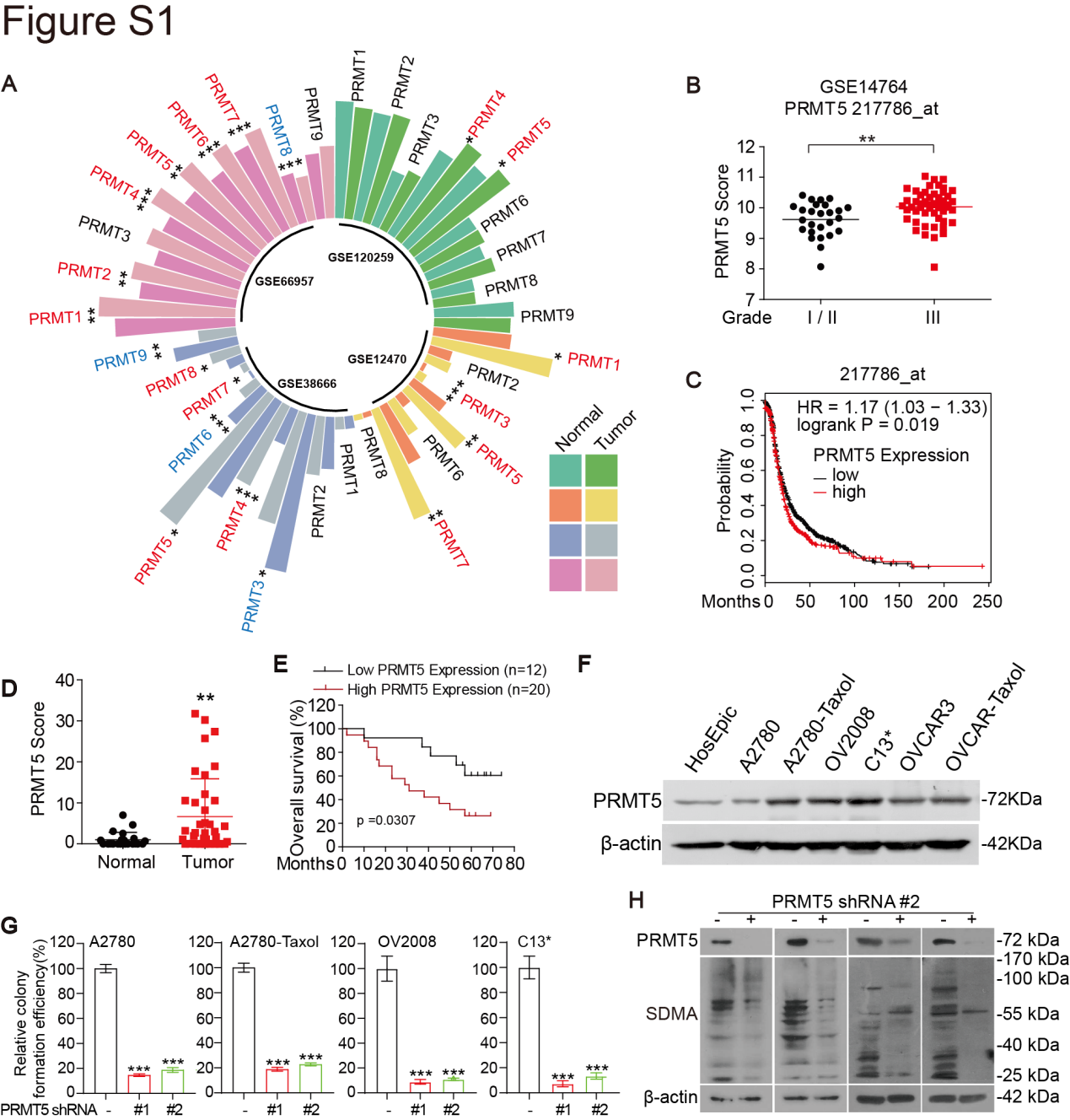
**

**Figure S1 PRMT5 is increased in ovarian cancer and important for ovarian cancer growth.**

**(A)** The expression of PRMT5 was examined based on GEO databases (GSE66957, GSE120259, GSE12470 and GSE38666). (**B)** The expression of PRMT5 was examined based on GEO databases (GSE14764). (**C)** Kaplan–Meier analysis of survival was performed according to the PRMT5 expression level in ovarian cancer. (**D**) The score of PRMT5 protein level were analyzed based on IHC. (**E**) Kaplan–Meier analysis of overall survival was performed on the basis of PRMT5 expression level in ovarian cancer patients (Cohort 2: ovarian cancer tissues [n=65]). (**F**) The expression of PRMT5 was detected in Taxol-resistant and corresponding parental ovarian cancer cell lines. (**G**) Statistical analyses of colony formation in Figure 1E were performed. (**H**) The PRMT5 expression and SDMA level were analyzed in all tumors by western blotting. (All error bars, mean values ± SD, p values were determined by unpaired two-tailed Student’s t test of n = 3 independent biological experiments. *p < 0.05; **p < 0.01; ***p < 0.001).


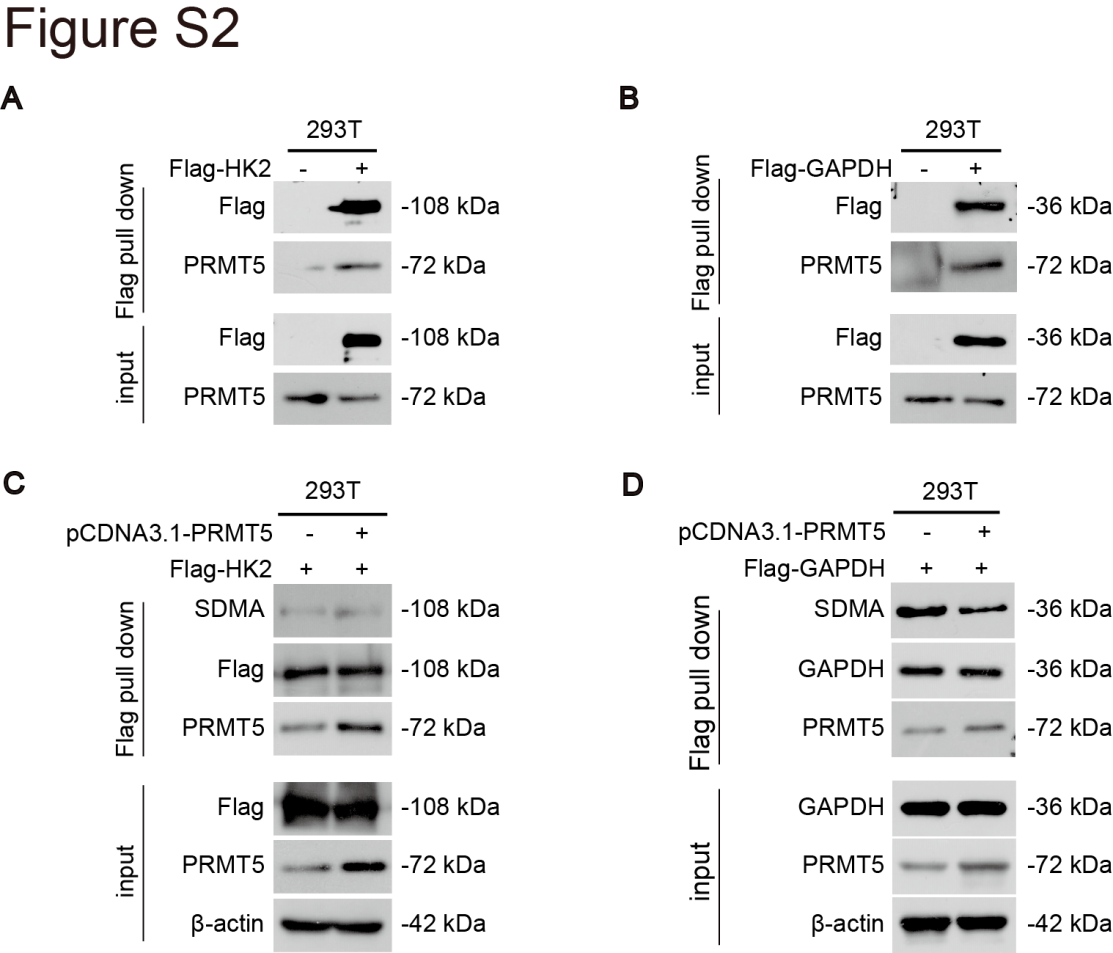


**Figure S2 Targeting PRMT5 decreases ENO1 activity mediated by arginine methylation.**

**(A)** The interaction between Flag-HK2 and endogenous PRMT5 was analyzed by Flag pull-down assays in 293T cells. (**B)** The interaction between Flag-GAPDH and endogenous PRMT5 was analyzed by Flag pull-down assays in 293T cells. (**C)** Flag pull-down of HK2 from HEK293T cells, which co-express Flag-HK2 with or without exogenous express untagged PRMT5, followed by western blot to detect the SDMA levels of HK2. (**D)** Flag pull-down of GAPDH from HEK293T cells, which co-express Flag-GAPDH with or without exogenous express untagged PRMT5, followed by western blot to detect the SDMA levels of GAPDH.


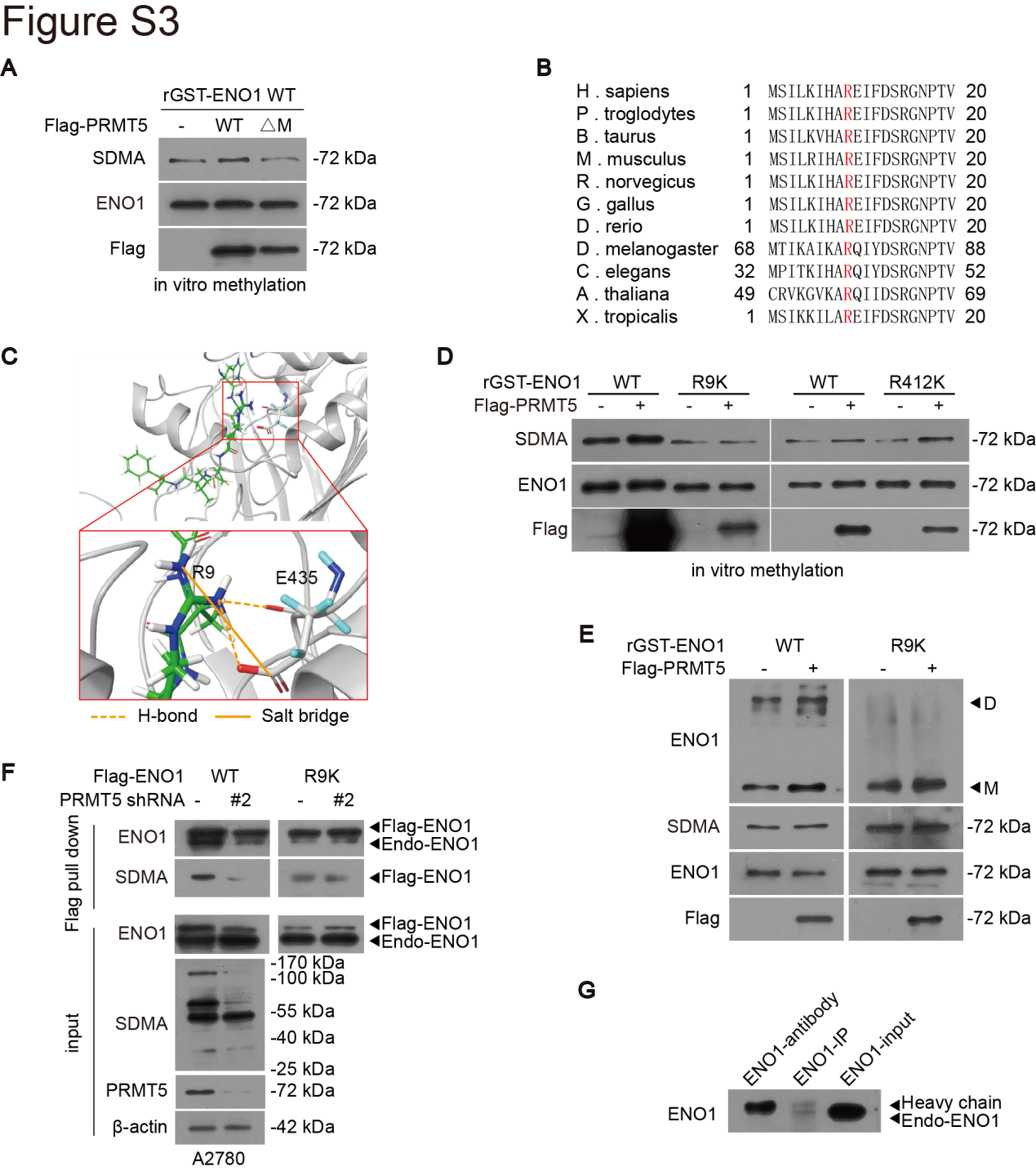


**Figure S3 PRMT5 signals high glucose condition and to increase ENO1 activity by methylating its R9 site.**

**(A)** *In vitro* methylation assay was performed to detect the SDMA level of purified rGST-ENO1, which was incubated with Flag-PRMT5 WT or inactivation mutant (△M), which were pulled down from 293T cells. (**B)** Sequence conservation analysis of ENO1 R9 site. (**C)** Interactions of ENO1 R9 with catalytic site of PRMT5. (**D)** *In vitro* methylation assay was performed to detect the SDMA level of purified rGST-ENO1, R9K, and R412K, which were incubated with or without Flag-PRMT5, which was pulled down from 293T cells. (**E)** Crosslinking assay was performed to detect dimeric and monomeric ENO1 (upper; ENO1 antibody). ENO1 protein input assessed by western blot is shown in the lower panel. (D, Dimeric GST-ENO1, 144KD; M, Monomeric GST-ENO1,72 KD). (**F)** The interaction between exogenous Flag-ENO1 and endogenous ENO1 was analyzed by Flag pull-down in control or PRMT5 knockdown A2780 cells, which exogenous express Flag-ENO1 WT or Flag-ENO1 R9K. (**G)** Western blottling was performed to localize heavy chain and endo-ENO1.


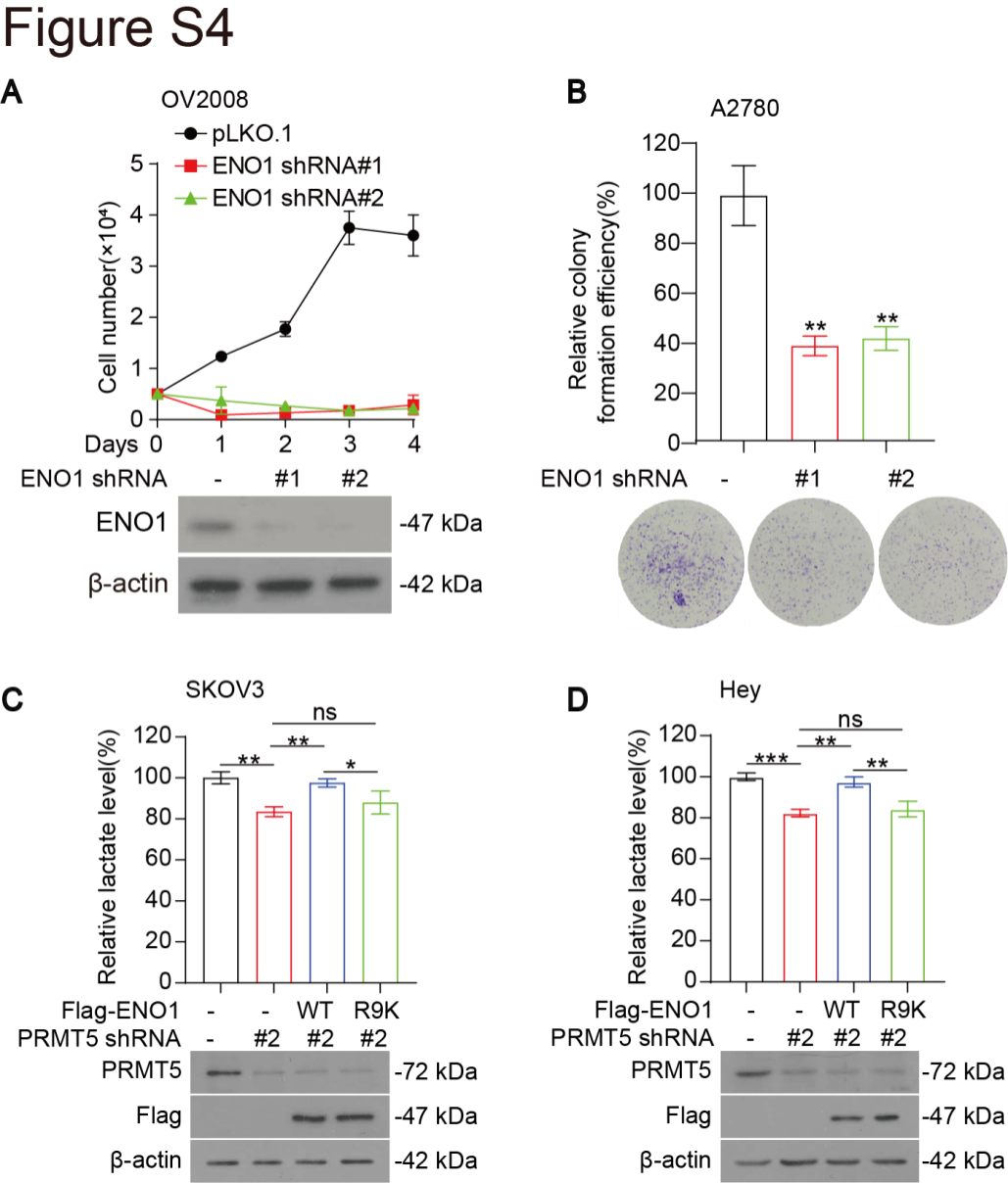


**Figure S4 PRMT5 promotes cancer cell growth and glycolysis flux through activating ENO1.**

**A** Cell proliferation was determined by cell number counting in ENO1 knockdown OV2008 cells. **B** Colony formation assay was determined in ENO1 knockdown A2780 cells. **C** Relative lactate level (upper) was determined by Lactic Acid assay kit in PRMT5 knockdown SKOV3 cells, which exogenous express with Flag-ENO1 WT or Flag-ENO1 R9K. **D** Relative lactate level (upper) was determined by Lactic Acid assay kit in PRMT5 knockdown Hey cells, which exogenous express with Flag-ENO1 WT or Flag-ENO1 R9K. (All error bars, mean values ± SD, p values were determined by unpaired two-tailed Student’s t test of n = 3 independent biological experiments. *p < 0.05; **< p < 0.01; ***p < 0.001).


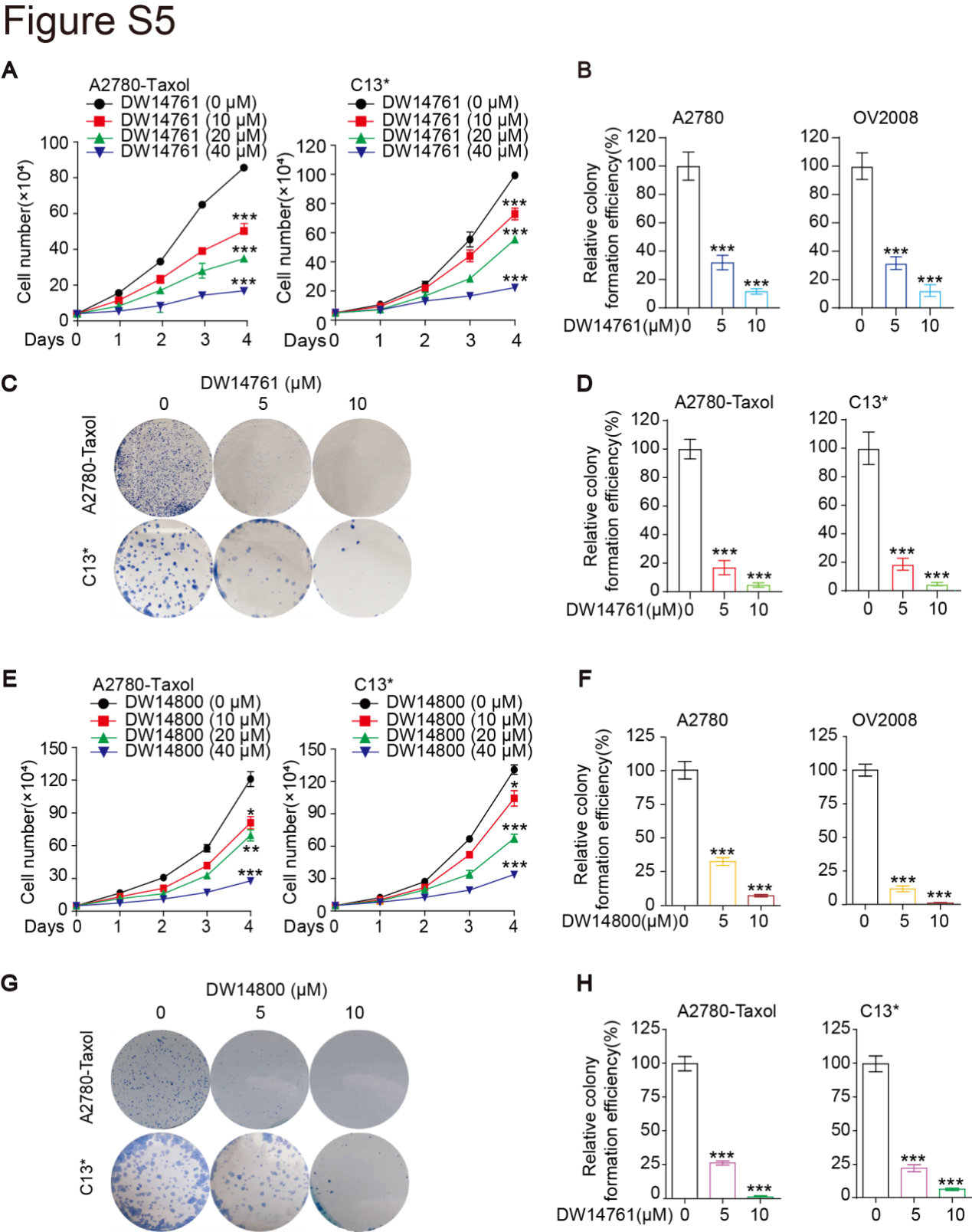


**Figure S5 PRMT5 inhibitors reduce ENO1 enzymatic activity and glycolytic pathway.**

(**A**) The cell proliferation was determined by cell number counting in ovarian cancer cells treated with different concentrations of DW14761. (**B**) Statistical analyses of colony formation in Figure 6B were performed. (**C**) Colony formation assay was determined in ovarian cancer cells treated with different concentrations of DW14761. (**D**) Statistical analyses of colony formation in Figure S5C were performed. (**E**) The cell proliferation was determined by cell number counting in ovarian cancer cells treated with different concentrations of DW14800. (**F**) Statistical analyses of colony formation in Figure 6D were performed. (**G**) Colony formation assay was determined in ovarian cancer cells treated with different concentrations of DW14800. (**H**) Statistical analyses of colony formation in Figure S5G were performed. (All error bars, mean values ± SD, p values were determined by unpaired two-tailed Student’s t test of n = 3 independent biological experiments. *< p < 0.05; **p < 0.01; ***p < 0.001).


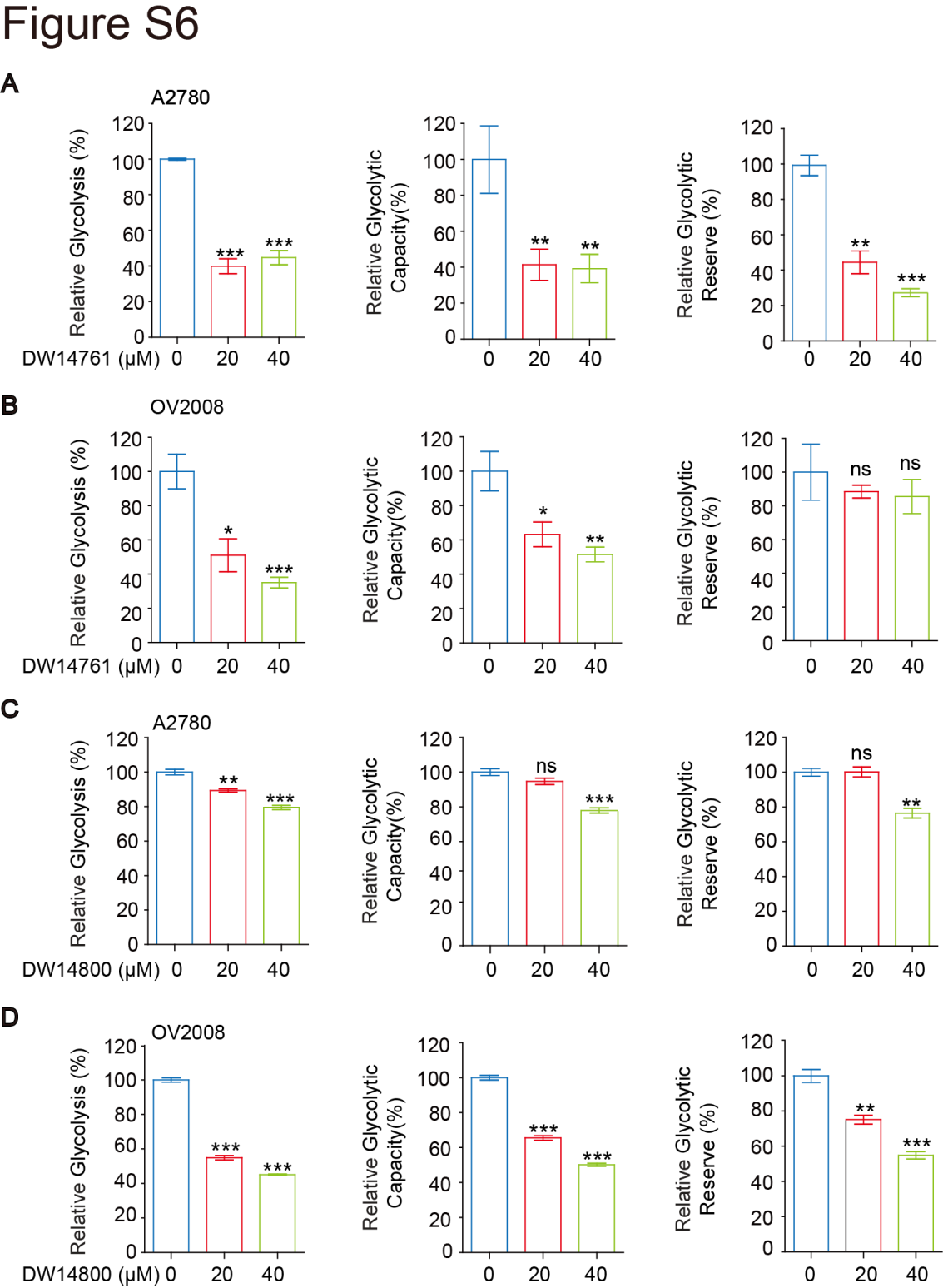


**Figure S6 PRMT5 inhibitors reduce glycolytic pathway.**

**(A)** Statistical analyses of ECAR measured in A2780 cells treated with DW14761 were performed. **(B)** Statistical analyses of ECAR measured in OV2008 cells treated with DW14761 were performed. **(C)** Statistical analyses of ECAR measured in A2780 cells treated with DW14800 were performed. **(D)** Statistical analyses of ECAR measured in OV2008 cells treated with DW14800 were performed. (All error bars, mean values ± SD, p values were determined by unpaired two-tailed Student’s t test of n = 3 independent biological experiments. *< p < 0.05; **p < 0.01; ***p < 0.001).


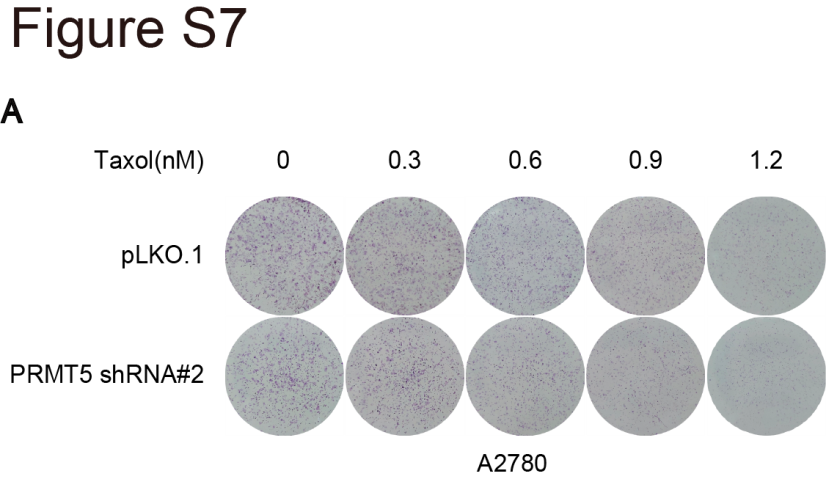


**Figure S7 Targeting PRMT5 enhances anti-tumor effect of Taxol in ovarian cancer**

**A** Colony formation assay was determined in PRMT5 knockdown or vector control A2780 cells treated with different concentrations of Taxol.

**Table S1. Expression of PRMT5 protein in ovarian cancer**

| **Variables** | **No. of case (*n*)** | **PRMT5**  **positive rate (%)** | ***χ^2^*** | ***P* value** |
| --- | --- | --- | --- | --- |
| Normal | 22 | 6(27.3) | 4.809 | 0.028 |
| Cancer | 39 | 22(56.4) |  |  |

**Table S2. Relationship between PRMT5 protein overexpression and the clinic pathological features of ovarian cancer**

| **Variables** | **No. of case (*n*)** | **PRMT5**  **positive rate (%)** | ***χ^2^*** | ***P* value** |
| --- | --- | --- | --- | --- |
| Age (years)  ＞47  ≤47 | 16  49 | 7(43.8)  22(44.9) | 0.006 | 0.936 |
| Tumor size  ≤10cm  ＞10cm | 24  41 | 14(58.3)  16(39.0) | 2.271 | 0.132 |
| Histological grade  Grade-1  Grade-2  Grade-3 | 8  30  17 | 5(62.5)  14(46.7)  9(52.9) | 0.674 | 0.714 |

**Table S3. KEY RESOURCES TABLE**

| **REAGENT or RESOURCE** | **SOURCE** | **IDENTIFIER** |
| --- | --- | --- |
| **Antibodies** |  |  |
| PRMT5 Rabbit PolyAb | proteintech | 18436-1-AP |
| ENO1 Rabbit PolyAb | proteintech | 11204-1-AP |
| PFKP Rabbit mAb | proteintech | [13389-1-AP](http://www.ptgcn.com/products/PFKP-Antibody-13389-1-AP.htm) |
| PGM1 | proteintech | 15161-1-AP |
| LDHA Rabbit mAb | proteintech | [19987-1-AP](http://www.ptgcn.com/products/LDHA-Specific-Antibody-19987-1-AP.htm) |
| PKM2 Rabbit Ab | proteintech | [15822-1-AP](http://www.ptgcn.com/products/PKM2-specific-Antibody-15822-1-AP.htm) |
| PGAM1 Rabbit PolyAb | proteintech | 16126-1-AP |
| GPI Rabbit PolyAb | proteintech | 15171-1-AP |
| HK2 Rabbit PolyAb | proteintech | 22029-1-AP |
| PGK1 Rabbit PolyAb | proteintech | 17811-1-AP |
| ALDOA Rabbit PolyAb | proteintech | 11217-1-AP |
| GAPDH Mouse MAb | proteintech | 60004-1-Ig |
| Bata Actin Mouse McAb | proteintech | 66009-1-Ig |
| Ki67 Rabbit PolyAb | Cell signaling technology | 9027S |
| Flag Rabbit PolyAb | proteintech | 20543-1-AP |
| Flag Mouse PolyAb | proteintech | 66008-3-Ig |
| [Symmetric Di-Methyl Arginine Motif [sdme-RG] MultiMab™ Rabbit mAb mix](https://www.cst-c.com.cn/products/primary-antibodies/symmetric-di-methyl-arginine-motif-sdme-rg-multimab-rabbit-mab-mix/13222?site-search-type=Products) | Cell signaling technology | 13222S |
| GST Antibody | Cell signaling technology | 2625S |

| \| **Bacterial and Virus Strains** \|  \|  \| \| --- \| --- \| --- \| \| Trans10 Chemically Competent Cell \| [TransGen Biotech](http://www.baidu.com/link?url=2lik0ADTVVBIG_Pn4V7hg4i7OGiZ9dMUqQ2Jzk3lPlFwz-SOqDvqkcZ8TmHxXHuhSZDDcbrhBCMMD-0RfRDnCq) \| Cat# CD101-01 \| \| *Trans* BL21(DE3) Chemically Competent Cell \| [TransGen Biotech](http://www.baidu.com/link?url=2lik0ADTVVBIG_Pn4V7hg4i7OGiZ9dMUqQ2Jzk3lPlFwz-SOqDvqkcZ8TmHxXHuhSZDDcbrhBCMMD-0RfRDnCq) \| Cat# CD601-03 \| \| **Chemicals, Peptides, and Recombinant Proteins** \| \| \| \| DW14761 \| This paper \| N/A \| \| Glucose \| Sigma \| G7021 \| \| 2-phosphoglycerate \| Shanghai yuanye \| S31738 \| \| ADP \| Sigma \| A2754 \| \| NADH \| Sigma \| N8129 \| \| PKM2/LDHA \| Sigma \| P0294 \| \| 3 × Flag peptide \| APExBIO \| A6001 \| \| Anti-Flag agarose affinity gel \| Sigma-Aldrich \| A4596 \| \| Protein A/G sepharose \| GE Healthcare Life Sciences \| 17-0618-01 \| \| Trizma base \| Sigma-Aldrich \| V900483 \| \| SAM \| Sigma \| A4377 \| \| puromycin \| InvivoGen \| Ant-pr-1 \| \| Polyethylenimine (PEI) \| Polysciences \| 23966 \| \| Polybrene \| Sigma \| H9268 \| \| TRIzol \| Thermo Fisher Scientific \| 15596018 \| \| Taxol \| Meilunbio \| MB1178 \| \| normal goat serum (NGS) \| ZSGB-BIO \| ZLI-9021 \| \| DAB \| MXB Biotechnologies \| DAB-0031/1031 \| \| Immobilon Western Chemiluminescent HRP Substrate \| Millipore \| WBKLS0500 \| \| Trypsin 0.5%EDTA \| Thermo Fisher Scientific \| 25200-072 \| \| DMEM Medium \| Thermo Fisher Scientific \| C11995500BT \| \| RPMI 1640 Medium \| Thermo Fisher Scientific \| C11875500BT \| \| RPMI 1640 Medium, no Phenol Red \| Thermo Fisher Scientific \| 11835-030 \| \| RPMI 1640 Medium, glucose-free \| Thermo Fisher Scientific \| 11879-020 \| \| **Critical Commercial Assays** \|  \|  \| \| TB Green® Premix Ex TaqTMⅡ(Tli RNaseH plus) \| TaKaRa \| RR820A \| \| Endo-free plasmid maxi kit \| Omega \| D6926-03 \| \| EasyPure plsmid miniprep kit \| [TransGen Biotech](http://www.baidu.com/link?url=2lik0ADTVVBIG_Pn4V7hg4i7OGiZ9dMUqQ2Jzk3lPlFwz-SOqDvqkcZ8TmHxXHuhSZDDcbrhBCMMD-0RfRDnCq) \| EM101-02 \| \| TransStart FastPfu DNA Polymerase \| [TransGen Biotech](http://www.baidu.com/link?url=2lik0ADTVVBIG_Pn4V7hg4i7OGiZ9dMUqQ2Jzk3lPlFwz-SOqDvqkcZ8TmHxXHuhSZDDcbrhBCMMD-0RfRDnCq) \| AP221-12 \| \| Fast mutagenesis system \| [TransGen Biotech](http://www.baidu.com/link?url=2lik0ADTVVBIG_Pn4V7hg4i7OGiZ9dMUqQ2Jzk3lPlFwz-SOqDvqkcZ8TmHxXHuhSZDDcbrhBCMMD-0RfRDnCq) \| FM111-01 \| \| Lactate Assay Kit \| Nanjing Jiancheng Bioengineering Institute \| A019-2-1 \| \| Seahorse XF glycolytic rate assay kit \| Seahorse Bioscience, Agilent Technologies \| [103344-100](https://www.chem.agilent.com/store/productDetail.jsp?catalogId=103344-100) \| \| Cell-LightTM Edu Apollo488 In Vitro Kit \| RIBOBIO \| C10310-3 \| \| **Experimental Models: Cell Lines** \|  \|  \| \| HEK293T \| This paper \| N/A \| \| HosEpic \| This paper \| N/A \| \| IOSE-80 \| This paper \| N/A \| \| OVCAR3 \| This paper \| N/A \| \| OVCAR3-Taxol \| This paper \| N/A \| \| SKOV3 \| This paper \| N/A \| \| Hey \| This paper \| N/A \| \| C13* \| University of Ottawa, Ontario, Canada \| Benjamin K. Tsang \| \| OV2008 \| University of Ottawa, Ontario, Canada \| Benjamin K. Tsang \| \| A2780 \| This paper \| N/A \| \| A2780-Taxol \| This paper \| N/A \| \| **shRNA** \|  \|  \| \| pLKO.1 \| TranSheepBio \| N/A \| \| PRMT5 shRNA #1 and #2 \| TranSheepBio \| N/A \| \| ENO1 shRNA #1 and #2 \| TranSheepBio \| N/A \| \| **siRNA** \|  \|  \| \| siRNA Negative control(PRMT5) \| GenePharma \| N/A \| \| siPRMT5 #1 \| GenePharma \| N/A \| \| siPRMT5 #2 \| GenePharma \| N/A \| \| **Recombinant DNA** \|  \|  \| \| pLVX3-PRMT5 \| This paper \| N/A \| \| pLVX3-PRMT5△M \| This paper \| N/A \| \| pLVX3-PRMT5-SM(silent mutation)#2 \| This paper \| N/A \| \| pLVX3-ENO1 \| This paper \| N/A \| \| pLVX3-GST-ENO1 \| This paper \| N/A \| \| pLVX3-ENO1 R9K \| This paper \| N/A \| \| pETM3C -PRMT5 \| This paper \| N/A \| \| pETM3C -PRMT5△M \| This paper \| N/A \| \| pETM3C-GST-ENO1 \| This paper \| N/A \| \| pETM3C-GST-ENO1 R9K \| This paper \| N/A \| \| pETM3C-GST-ENO1 R15K \| This paper \| N/A \| \| pETM3C-GST-ENO1 R50K \| This paper \| N/A \| \| pETM3C-GST-ENO1 R183K \| This paper \| N/A \| \| pETM3C-GST-ENO1 R372K \| This paper \| N/A \| \| pETM3C-GST-ENO1 R412K \| This paper \| N/A \| \| **Oligonucleotides** \|  \|  \| \| PRMT5-F \| 5'-CGCGGATCCATGGCGGCGATGGCGGTC-3' \| N/A \| \| PRMT5-R \| 5'-CCGCTCGAGCTAGAGGCCAATGGTATATGAGCGGCCTG-3' \| N/A \| \| PRMT5-SM#2-F \| 5'-TGACCCTGAGGCCCAGTTCGAAATGCCTTAT-3' \| N/A \| \| PRMT5-SM#2-R \| 5'-TTCGAACTGGGCCTCAGGGTCACGGTCCTTC-3' \| N/A \| \| PRMT5-△M-F \| 5'-GGTACTGATGGTGCTGCCCCTGGTGAACGCTTCCCT-3' \| N/A \| \| PRMT5-△M-R \| 5'-AGCGTTCACCAGGGGCAGCACCATCAGTACCTGGACAT-3' \| N/A \| \| PRMT5-qPCR -F \| 5'- CTGTCTTCCATCCGCGTTTCA-3' \| N/A \| \| PRMT5-qPCR -R \| 5'- GCAGTAGGTCTGATCGTGTCTG -3' \| N/A \| \| ENO1-R9K-F \| 5'-CTCAAGATCCATGCCAAGGAGATCTTT-3' \| N/A \| \| ENO1-R9K-R \| 5'-TTGGCATGGATCTTGAGAATAGACAT-3' \| N/A \| \| ENO1-R15K-F \| 5'-GAGATCTTTGACTCTAAAGGGAATCCCA-3' \| N/A \| \| ENO1-R15K-R \| 5'-TTTAGAGTCAAAGATCTCCCTGGCATGG -3' \| N/A \| \| ENO1-R50K-F \| 5'-GAGGCCCTAGAGCTCAAGGACAATGAT -3' \| N/A \| \| ENO1-R50K-R \| 5'-TTGAGCTCTAGGGCCTCATAGATAC-3' \| N/A \| \| ENO1-R183K-F \| 5'-TTCAGGGAAGCCATGAAGATTGGAGCAG-3 \| N/A \| \| ENO1-R183K-R \| 5'-CTTCATGGCTTCCCTGAAGTTTGCTGCA-3' \| N/A \| \| ENO1-R372K-F \| 5'-GTCATGGTGTCTCATAAGTCGGGGGAGA-3' \| N/A \| \| ENO1-R372K-R \| 5'-CTTATGAGACACCATGACGCCCCAACCA-3' \| N/A \| \| ENO1-R412K-F \| 5'-ACAACCAGCTCCTCAAAATTGAAGAG-3' \| N/A \| \| ENO1-R412K-R \| 5'-TTGAGGAGCTGGTTGTACTTGGCCAA -3' \| N/A \| \| siRNA Negative control(PRMT5) \| 5'-UUCUCCGAACGUGUCACGUTT-3' \| N/A \| \| siPRMT5 #1 \| 5'-UCAAGAACUCCCUGGAAUATT-3' \| N/A \| \| siPRMT5 #2 \| 5'-CCAGUUUGAGAUGCCUUAUTT-3' \| N/A \| \| PRMT5 shRNA #1 \| 5'-CCGGCCTCAAGAACTCCCTGGAATACTCGAGTATTCCAGGGAGTTCTTGAGGTTTTTG-3' \| N/A \| \| PRMT5 shRNA #2 \| 5'-CCGGGCCCAGTTTGAGATGCCTTATCTCGAGATAAGGCATCTCAAACTGGGCTTTTTG-3' \| N/A \| \| ENO1 shRNA #1 \| 5'-CCGGCGTACCGCTTCCTTAGAACTTCTCGAGAAGTTCTAAGGAAGCGGTACGTTTTT-3' \| N/A \| \| ENO1 shRNA #2 \| 5'-CCGGCCGGCGTTCAATGTCATCAATCTCGAGATTGATGACATTGAACGCCGGTTTTT-3' \| N/A \| \| **Software and Algorithms** \|  \|  \| \| GraphPad Prism 5 \| Graph- Pad \| Graph- Pad \| \| Adobe Illustrator \| Adobe \| Adobe \| \| ImageJ \| National Institutes of Health \| National Institutes of Health \| \| Leica Application Suite X -2.0.1 \| Leica Microsystems \| N/A \| \| Compusyn \| Chou TC.2010 \| N/A \| |
| --- | --- | --- | --- | --- | --- | --- | --- | --- | --- | --- | --- | --- | --- | --- | --- | --- | --- | --- | --- | --- | --- | --- | --- | --- | --- | --- | --- | --- | --- | --- | --- | --- | --- | --- | --- | --- | --- | --- | --- | --- | --- | --- | --- | --- | --- | --- | --- | --- | --- | --- | --- | --- | --- | --- | --- | --- | --- | --- | --- | --- | --- | --- | --- | --- | --- | --- | --- | --- | --- | --- | --- | --- | --- | --- | --- | --- | --- | --- | --- | --- | --- | --- | --- | --- | --- | --- | --- | --- | --- | --- | --- | --- | --- | --- | --- | --- | --- | --- | --- | --- | --- | --- | --- | --- | --- | --- | --- | --- | --- | --- | --- | --- | --- | --- | --- | --- | --- | --- | --- | --- | --- | --- | --- | --- | --- | --- | --- | --- | --- | --- | --- | --- | --- | --- | --- | --- | --- | --- | --- | --- | --- | --- | --- | --- | --- | --- | --- | --- | --- | --- | --- | --- | --- | --- | --- | --- | --- | --- | --- | --- | --- | --- | --- | --- | --- | --- | --- | --- | --- | --- | --- | --- | --- | --- | --- | --- | --- | --- | --- | --- | --- | --- | --- | --- | --- | --- | --- | --- | --- | --- | --- | --- | --- | --- | --- | --- | --- | --- | --- | --- | --- | --- | --- | --- | --- | --- | --- | --- | --- | --- | --- | --- | --- | --- | --- | --- | --- | --- | --- | --- | --- | --- | --- | --- | --- | --- | --- | --- | --- | --- | --- | --- | --- | --- | --- | --- | --- | --- | --- | --- | --- | --- | --- | --- | --- | --- | --- | --- | --- | --- | --- | --- | --- | --- | --- | --- | --- | --- | --- | --- | --- | --- | --- | --- | --- | --- | --- | --- | --- | --- | --- | --- | --- | --- | --- | --- | --- | --- | --- | --- | --- | --- | --- | --- | --- | --- | --- | --- | --- | --- | --- | --- | --- | --- | --- | --- | --- | --- | --- | --- | --- | --- | --- | --- | --- | --- | --- | --- | --- | --- | --- | --- | --- | --- | --- | --- | --- | --- | --- | --- | --- |
